# Supplementary material for: Spatiotemporal variability in dengue transmission intensity in Jakarta, Indonesia
Source: PLoS Negl Trop Dis. 2020 Mar 6;14(3):e0008102. doi: 10.1371/journal.pntd.0008102 (PMC7080271; doi:10.1371/journal.pntd.0008102)
Supplement: S1 Text — (DOCX) [file pntd.0008102.s001.docx]

**Supplementary Information**

**Spatiotemporal Variability in Dengue Transmission Intensity in Jakarta, Indonesia.**

Megan O’Driscoll^1^, Natsuko Imai^1^, Neil M. Ferguson^1^, Sri Rezeki Hadinegoro^2^, Hindra Irawan Satari^2^, Clarence C. Tam^3,4^, Ilaria Dorigatti^1^

^1^MRC Centre for Global Infectious Disease Analysis, Imperial College London, London, United Kingdom

^2^Department of Child Health, Faculty of Medicine Universitas Indonesia, Jakarta, Indonesia

^3^Saw Swee Hock School of Public Health, National University of Singapore and National University Health System, Singapore

^4^London School of Hygiene & Tropical Medicine, London, United Kingdom

Corresponding author: [m.odriscoll@imperial.ac.uk](mailto:m.odriscoll@imperial.ac.uk)

Contents

[**1.** **DHF case-notification data reported in Jakarta.** 3](#_Toc522278809)

[**2.** **Population age-structures** 6](#_Toc522278810)

[**3.** **Mathematical Models** 8](#_Toc522278811)

[3.1 Incidence Model 8](#_Toc522278812)

[3.2 Likelihood function 8](#_Toc522278813)

[3.3 Seropositive proportion of the population 9](#_Toc522278814)

[3.4 Basic Reproduction Number 9](#_Toc522278815)

[3.5 Linear regression for prediction of force of infection 10](#_Toc522278816)

[**4.** **Results** 11](#_Toc522278817)

[4.1 Jakarta province: parameter estimates and model fits from yearly and cumulative data 11](#_Toc522278818)

[4.2 Jakarta subdistricts: parameter estimates and model fits from cumulative data 13](#_Toc522278819)

[4.3 Maps of subdistrict locations, spatial autocorrelation & annual transmission intensity 30](#_Toc522278820)

[5. References 33](#_Toc522278821)

## **DHF case-notification data reported in Jakarta.**

**Table A. Annual number of hospitalized DHF cases per subdistrict between 2008 and 2017, excluding cases in infants <1 year. Available online at (1).**

| **Subdistrict** | **2008** | **2009** | **2010** | **2011** | **2012** | **2013** | **2014** | **2015** | **2016** | **2017** | **2008-2017** |
| --- | --- | --- | --- | --- | --- | --- | --- | --- | --- | --- | --- |
| Cakung | 1,608 | 1,618 | 1,353 | 439 | 494 | 1,137 | 611 | 462 | 1,556 | 434 | 9,712 |
| Cempaka Putih | 602 | 640 | 586 | 187 | 160 | 221 | 188 | 133 | 445 | 100 | 3,262 |
| Cengkareng | 704 | 378 | 1,176 | 367 | 453 | 676 | 856 | 592 | 3157 | 418 | 8,777 |
| Cilandak | 764 | 705 | 692 | 187 | 262 | 407 | 406 | 178 | 502 | 56 | 4,159 |
| Cilincing | 535 | 1,040 | 730 | 390 | 368 | 412 | 404 | 464 | 975 | 280 | 5,598 |
| Cipayung | 467 | 520 | 677 | 225 | 314 | 542 | 446 | 284 | 1227 | 225 | 4,927 |
| Ciracas | 478 | 657 | 535 | 217 | 333 | 451 | 416 | 257 | 1071 | 196 | 4,611 |
| Duren Sawit | 2,015 | 1,557 | 1,595 | 677 | 655 | 1,113 | 911 | 639 | 1905 | 499 | 11,566 |
| Gambir | 272 | 196 | 239 | 99 | 112 | 146 | 150 | 102 | 307 | 62 | 1,685 |
| Grogol Petamburan | 620 | 444 | 482 | 163 | 288 | 460 | 622 | 257 | 794 | 135 | 4,265 |
| Jagakarsa | 674 | 834 | 754 | 207 | 398 | 650 | 664 | 414 | 1,642 | 277 | 6,514 |
| Jatinegara | 960 | 842 | 877 | 274 | 282 | 528 | 456 | 229 | 1,196 | 331 | 5,975 |
| Johar Baru | 405 | 375 | 538 | 134 | 150 | 139 | 153 | 86 | 360 | 80 | 2,420 |
| Kali Deres | 383 | 181 | 540 | 224 | 303 | 409 | 530 | 379 | 1,820 | 251 | 5,020 |
| Kebayoran Baru | 430 | 516 | 505 | 161 | 240 | 356 | 395 | 247 | 810 | 149 | 3,809 |
| Kebayoran Lama | 961 | 871 | 894 | 345 | 428 | 447 | 527 | 288 | 1127 | 264 | 6,152 |
| Kebun Jeruk | 911 | 764 | 749 | 200 | 191 | 218 | 260 | 130 | 432 | 92 | 3,947 |
| Kelapa Gading | 712 | 1,210 | 856 | 448 | 371 | 1,311 | 1,248 | 1,452 | 1,093 | 191 | 8,892 |
| Kemayoran | 772 | 782 | 845 | 303 | 294 | 518 | 583 | 303 | 795 | 151 | 5,346 |
| Kembangan | 399 | 437 | 704 | 207 | 253 | 415 | 519 | 192 | 641 | 106 | 3,873 |
| Kep. Seribu Selatan | 0 | 2 | 2 | 12 | 8 | 4 | 8 | 13 | 10 | 5 | 64 |
| Kep. Seribu Utara | 1 | 5 | 1 | 0 | 4 | 0 | 3 | 3 | 7 | 0 | 24 |
| Koja | 737 | 1,137 | 896 | 488 | 386 | 377 | 443 | 385 | 1,153 | 290 | 6,292 |
| Kramat Jati | 810 | 643 | 786 | 258 | 332 | 495 | 481 | 253 | 1,188 | 237 | 5,483 |
| Makasar | 537 | 458 | 523 | 228 | 269 | 359 | 389 | 261 | 793 | 228 | 4,045 |
| Mampang Prapatan | 616 | 552 | 431 | 105 | 154 | 311 | 299 | 146 | 638 | 114 | 3,366 |
| Matraman | 681 | 706 | 736 | 211 | 198 | 258 | 225 | 153 | 659 | 130 | 3,957 |
| Menteng | 331 | 220 | 254 | 91 | 95 | 126 | 112 | 62 | 228 | 54 | 1,573 |
| Pademangan | 301 | 362 | 391 | 213 | 198 | 272 | 238 | 127 | 552 | 109 | 2,763 |
| Palmerah | 711 | 565 | 429 | 258 | 360 | 457 | 255 | 154 | 586 | 168 | 3,943 |
| Pancoran | 663 | 716 | 655 | 120 | 192 | 409 | 357 | 233 | 557 | 97 | 3,999 |
| Pasar Minggu | 1,383 | 1,257 | 1,070 | 318 | 408 | 634 | 787 | 337 | 1,383 | 322 | 7,899 |
| Pasar Rebo | 327 | 490 | 451 | 288 | 388 | 417 | 348 | 176 | 926 | 207 | 4,018 |
| Penjaringan | 596 | 592 | 522 | 314 | 331 | 556 | 571 | 169 | 1104 | 146 | 4,901 |
| Pesanggrahan | 305 | 419 | 448 | 120 | 175 | 339 | 388 | 176 | 722 | 109 | 3,201 |
| Pulo Gadung | 959 | 1,089 | 827 | 271 | 436 | 793 | 482 | 344 | 1,454 | 428 | 7,083 |
| Sawah Besar | 332 | 325 | 373 | 170 | 149 | 174 | 162 | 101 | 466 | 75 | 2,327 |
| Senen | 452 | 385 | 392 | 127 | 115 | 194 | 137 | 69 | 284 | 40 | 2,195 |
| Setiabudi | 382 | 335 | 367 | 164 | 135 | 244 | 166 | 65 | 440 | 74 | 2,372 |
| Taman Sari | 314 | 325 | 347 | 139 | 143 | 157 | 157 | 75 | 318 | 37 | 2,012 |
| Tambora | 428 | 366 | 375 | 181 | 207 | 252 | 282 | 221 | 531 | 127 | 2,970 |
| Tanah Abang | 233 | 200 | 311 | 133 | 175 | 226 | 210 | 105 | 484 | 98 | 2,175 |
| Tanjung Priok | 1,109 | 1,135 | 1,286 | 616 | 455 | 746 | 627 | 564 | 1,525 | 307 | 8,370 |
| Tebet | 776 | 843 | 901 | 273 | 239 | 492 | 444 | 214 | 812 | 171 | 5,165 |
| *Total Cases in Jakarta* | *27,656* | *27,694* | *28,101* | *10,552* | *11,901* | *18,848* | *17,916* | *11,494* | *38,675* | *7,870* | *200,707* |

**Table B. Annual subdistrict incidence rates (IR) per 1,000 population, between 2008 and 2017, excluding cases in infants <1 year. Case data are available online at (1). Calculated annual age-structures are reported in Supplementary File S2.**

| **Subdistrict** | **2008** | **2009** | **2010** | **2011** | **2012** | **2013** | **2014** | **2015** | **2016** | **2017** | **2008-2017** |
| --- | --- | --- | --- | --- | --- | --- | --- | --- | --- | --- | --- |
| Cakung | 3.610 | 3.632 | 3.037 | 0.964 | 1.062 | 2.395 | 1.261 | 0.917 | 3.071 | 0.857 | 2.081 |
| Cempaka Putih | 7.981 | 8.484 | 7.769 | 2.326 | 1.875 | 2.447 | 1.973 | 1.398 | 4.459 | 1.002 | 3.971 |
| Cengkareng | 1.459 | 0.784 | 2.438 | 0.756 | 0.927 | 1.375 | 1.730 | 1.189 | 6.137 | 0.813 | 1.761 |
| Cilandak | 4.014 | 3.704 | 3.636 | 0.967 | 1.334 | 2.040 | 2.004 | 0.873 | 2.428 | 0.271 | 2.127 |
| Cilincing | 1.438 | 2.794 | 1.961 | 1.041 | 0.976 | 1.085 | 1.057 | 1.187 | 2.432 | 0.698 | 1.467 |
| Cipayung | 2.157 | 2.402 | 3.128 | 1.016 | 1.386 | 2.340 | 1.885 | 1.149 | 4.831 | 0.886 | 2.118 |
| Ciracas | 1.924 | 2.645 | 2.154 | 0.853 | 1.280 | 1.696 | 1.530 | 0.920 | 3.816 | 0.698 | 1.752 |
| Duren Sawit | 5.598 | 4.326 | 4.431 | 1.835 | 1.734 | 2.878 | 2.302 | 1.575 | 4.700 | 1.231 | 3.061 |
| Gambir | 3.474 | 2.503 | 3.052 | 1.185 | 1.262 | 1.553 | 1.511 | 1.029 | 3.070 | 0.620 | 1.926 |
| Grogol Petamburan | 2.858 | 2.047 | 2.222 | 0.743 | 1.300 | 2.055 | 2.750 | 1.142 | 3.551 | 0.604 | 1.927 |
| Jagakarsa | 2.394 | 2.962 | 2.678 | 0.725 | 1.374 | 2.213 | 2.230 | 1.332 | 5.159 | 0.870 | 2.194 |
| Jatinegara | 3.437 | 3.014 | 3.140 | 0.957 | 0.961 | 1.757 | 1.483 | 0.734 | 3.856 | 1.067 | 2.041 |
| Johar Baru | 3.730 | 3.454 | 4.956 | 1.160 | 1.224 | 1.073 | 1.121 | 0.623 | 2.499 | 0.555 | 2.040 |
| Kali Deres | 1.029 | 0.486 | 1.451 | 0.599 | 0.805 | 1.081 | 1.393 | 0.968 | 4.668 | 0.644 | 1.312 |
| Kebayoran Baru | 3.100 | 3.720 | 3.640 | 1.141 | 1.672 | 2.439 | 2.663 | 0.827 | 5.419 | 0.997 | 2.562 |
| Kebayoran Lama | 3.429 | 3.108 | 3.190 | 1.212 | 1.481 | 1.524 | 1.771 | 1.935 | 3.686 | 0.864 | 2.220 |
| Kebun Jeruk | 2.977 | 2.496 | 2.447 | 0.648 | 0.614 | 0.695 | 0.822 | 0.405 | 1.329 | 0.283 | 1.271 |
| Kelapa Gading | 5.749 | 9.771 | 6.912 | 3.572 | 2.922 | 10.199 | 9.592 | 11.039 | 8.213 | 1.435 | 6.941 |
| Kemayoran | 3.960 | 4.011 | 4.334 | 1.459 | 1.334 | 2.223 | 2.372 | 1.208 | 3.104 | 0.590 | 2.460 |
| Kembangan | 1.636 | 1.791 | 2.886 | 0.842 | 1.021 | 1.661 | 2.062 | 0.732 | 2.426 | 0.401 | 1.546 |
| Kep. Seribu Selatan | 0.000 | 0.239 | 0.239 | 1.367 | 0.869 | 0.416 | 0.796 | 1.271 | 0.911 | 0.455 | 0.656 |
| Kep. Seribu Utara | 0.080 | 0.399 | 0.080 | 0.000 | 0.290 | 0.000 | 0.199 | 0.196 | 0.436 | 0.000 | 0.168 |
| Koja | 2.450 | 3.780 | 2.979 | 1.610 | 1.264 | 1.225 | 1.429 | 1.207 | 3.592 | 0.903 | 2.044 |
| Kramat Jati | 3.144 | 2.496 | 3.051 | 0.978 | 1.230 | 1.792 | 1.704 | 0.876 | 4.126 | 0.823 | 2.022 |
| Makasar | 2.947 | 2.513 | 2.870 | 1.222 | 1.408 | 1.837 | 1.947 | 1.265 | 3.858 | 1.109 | 2.098 |
| Mampang Prapatan | 4.419 | 3.960 | 3.092 | 0.742 | 1.073 | 2.137 | 2.026 | 0.974 | 4.196 | 0.750 | 2.337 |
| Matraman | 4.146 | 4.299 | 4.481 | 1.252 | 1.146 | 1.457 | 1.241 | 0.837 | 3.596 | 0.709 | 2.316 |
| Menteng | 4.761 | 3.164 | 3.653 | 1.227 | 1.205 | 1.510 | 1.271 | 0.699 | 2.472 | 0.585 | 2.055 |
| Pademangan | 1.969 | 2.368 | 2.558 | 1.381 | 1.272 | 1.732 | 1.502 | 0.785 | 3.443 | 0.680 | 1.769 |
| Palmerah | 3.434 | 2.728 | 2.072 | 1.234 | 1.707 | 2.147 | 1.187 | 0.710 | 2.721 | 0.780 | 1.872 |
| Pancoran | 4.526 | 4.888 | 4.472 | 0.807 | 1.273 | 2.673 | 2.300 | 1.463 | 3.466 | 0.604 | 2.647 |
| Pasar Minggu | 4.965 | 4.512 | 3.841 | 1.125 | 1.423 | 2.181 | 2.670 | 1.122 | 4.555 | 1.061 | 2.745 |
| Pasar Rebo | 1.770 | 2.652 | 2.441 | 1.523 | 2.006 | 2.109 | 1.722 | 0.847 | 4.414 | 0.987 | 2.047 |
| Penjaringan | 2.100 | 2.086 | 1.839 | 1.096 | 1.144 | 1.905 | 1.938 | 0.554 | 3.699 | 0.489 | 1.685 |
| Pesanggrahan | 1.425 | 1.958 | 2.093 | 0.552 | 0.794 | 1.516 | 1.711 | 0.757 | 3.023 | 0.456 | 1.429 |
| Pulo Gadung | 3.659 | 4.155 | 3.156 | 1.009 | 1.584 | 2.813 | 1.671 | 1.187 | 4.972 | 1.464 | 2.567 |
| Sawah Besar | 3.183 | 3.116 | 3.576 | 1.529 | 1.262 | 1.392 | 1.228 | 0.766 | 3.507 | 0.564 | 2.012 |
| Senen | 4.612 | 3.929 | 4.000 | 1.216 | 1.038 | 1.655 | 1.108 | 0.560 | 2.199 | 0.310 | 2.063 |
| Setiabudi | 3.650 | 3.201 | 3.507 | 1.542 | 1.250 | 2.224 | 1.490 | 0.585 | 3.873 | 0.651 | 2.197 |
| Taman Sari | 2.622 | 2.714 | 2.897 | 1.146 | 1.164 | 1.262 | 1.247 | 0.598 | 2.557 | 0.298 | 1.650 |
| Tambora | 1.684 | 1.440 | 1.475 | 0.705 | 0.799 | 0.964 | 1.069 | 0.837 | 2.041 | 0.488 | 1.150 |
| Tanah Abang | 1.708 | 1.466 | 2.280 | 0.916 | 1.136 | 1.387 | 1.223 | 0.614 | 2.678 | 0.542 | 1.395 |
| Tanjung Priok | 2.997 | 3.068 | 3.476 | 1.650 | 1.208 | 1.963 | 1.636 | 1.456 | 3.886 | 0.782 | 2.212 |
| Tebet | 3.608 | 3.920 | 4.190 | 1.248 | 1.075 | 2.176 | 1.932 | 0.925 | 3.452 | 0.727 | 2.325 |
| *Average IR in Jakarta* | *2.980* | *2.984* | *3.027* | *1.115* | *1.234* | *1.917* | *1.789* | *1.128* | *3.753* | *0.764* | *2.069* |

## **Population age-structures**

**Table C. Average population age-structures calculated for the period 2008-2017. Yearly population age-structures used are given in Supplementary file S2.**

| **Subdistrict** | **1-4** | **5-9** | **10-14** | **15-19** | **20-44** | **45-54** | **55-64** | **65-74** | **>75** | **Total** |
| --- | --- | --- | --- | --- | --- | --- | --- | --- | --- | --- |
| Cakung | 43,697 | 44,254 | 38,036 | 36,500 | 223,198 | 51,694 | 26,377 | 7,617 | 1,934 | 473,307 |
| Cempaka Putih | 6,458 | 6,957 | 6,772 | 6,810 | 37,785 | 11,736 | 6,295 | 3,118 | 1,302 | 87,233 |
| Cengkareng | 44,150 | 43,581 | 37,950 | 39,562 | 236,817 | 54,006 | 26,165 | 8,674 | 2,531 | 493,436 |
| Cilandak | 15,313 | 16,317 | 15,381 | 15,427 | 88,278 | 25,632 | 13,151 | 6,077 | 2,464 | 198,040 |
| Cilincing | 35,918 | 35,656 | 31,501 | 31,366 | 182,056 | 37,736 | 19,207 | 7,011 | 1,847 | 382,298 |
| Cipayung | 20,706 | 21,032 | 19,130 | 18,701 | 105,033 | 27,634 | 13,895 | 4,665 | 1,288 | 232,084 |
| Ciracas | 23,107 | 23,194 | 20,578 | 20,496 | 120,456 | 31,309 | 17,573 | 5,633 | 1,453 | 263,799 |
| Duren Sawit | 31,740 | 32,991 | 29,257 | 28,360 | 172,836 | 44,272 | 27,216 | 12,582 | 3,275 | 382,529 |
| Gambir | 6,119 | 6,612 | 6,606 | 6,839 | 38,769 | 11,940 | 7,714 | 3,595 | 1,771 | 89,965 |
| Grogol Petamburan | 17,173 | 16,753 | 14,119 | 15,488 | 103,285 | 26,255 | 16,376 | 8,730 | 3,202 | 221,381 |
| Jagakarsa | 26,108 | 26,692 | 23,462 | 23,584 | 134,865 | 34,350 | 17,986 | 6,907 | 1,906 | 295,860 |
| Jatinegara | 24,706 | 25,004 | 22,471 | 22,690 | 130,494 | 36,601 | 21,121 | 9,337 | 3,383 | 295,807 |
| Johar Baru | 9,943 | 10,991 | 10,365 | 10,238 | 55,403 | 15,359 | 8,449 | 3,593 | 1,260 | 125,601 |
| Kali Deres | 33,901 | 34,381 | 30,143 | 32,642 | 181,921 | 41,197 | 17,602 | 6,013 | 1,890 | 379,690 |
| Kebayoran Baru | 11,953 | 12,669 | 12,044 | 12,291 | 69,573 | 21,463 | 11,799 | 5,221 | 2,268 | 159,281 |
| Kebayoran Lama | 22,318 | 22,909 | 20,557 | 21,168 | 125,618 | 34,262 | 18,443 | 8,511 | 2,763 | 276,549 |
| Kebun Jeruk | 27,209 | 25,284 | 22,077 | 23,838 | 147,968 | 35,568 | 20,546 | 8,723 | 2,758 | 313,971 |
| Kelapa Gading | 9,263 | 9,405 | 8,310 | 9,123 | 57,593 | 16,028 | 11,222 | 5,427 | 1,653 | 128,024 |
| Kemayoran | 17,748 | 19,207 | 18,418 | 17,810 | 98,254 | 28,287 | 15,698 | 7,412 | 2,638 | 225,472 |
| Kembangan | 21,776 | 21,294 | 18,059 | 19,848 | 118,779 | 28,145 | 16,102 | 5,972 | 1,810 | 251,785 |
| Kep. Seribu Selatan | 890 | 953 | 981 | 817 | 4,021 | 1,005 | 513 | 245 | 66 | 9,491 |
| Kep. Seribu Utara | 1,327 | 1,349 | 1,500 | 1,348 | 5,876 | 1,550 | 768 | 290 | 143 | 14,151 |
| Koja | 28,869 | 27,723 | 23,866 | 24,607 | 145,298 | 33,302 | 17,185 | 6,378 | 1,751 | 308,979 |
| Kramat Jati | 24,057 | 24,246 | 21,089 | 20,756 | 123,257 | 32,979 | 17,576 | 6,881 | 2,166 | 273,007 |
| Makasar | 16,706 | 16,885 | 14,949 | 14,980 | 87,556 | 23,820 | 12,792 | 4,700 | 1,300 | 193,688 |
| Mampang Prapatan | 12,060 | 12,351 | 10,963 | 11,293 | 66,329 | 18,069 | 9,168 | 3,628 | 1,155 | 145,016 |
| Matraman | 13,950 | 14,394 | 13,230 | 13,202 | 75,391 | 23,547 | 12,758 | 5,565 | 2,147 | 174,184 |
| Menteng | 5,867 | 6,225 | 6,238 | 6,483 | 34,212 | 10,819 | 6,723 | 2,804 | 1,256 | 80,627 |
| Pademangan | 13,037 | 13,311 | 11,556 | 11,955 | 73,183 | 18,063 | 9,953 | 4,239 | 1,373 | 156,670 |
| Palmerah | 17,718 | 17,749 | 15,308 | 16,755 | 96,943 | 25,674 | 13,555 | 5,760 | 2,168 | 211,630 |
| Pancoran | 12,890 | 12,888 | 11,157 | 11,345 | 69,903 | 18,787 | 9,758 | 4,615 | 1,441 | 152,784 |
| Pasar Minggu | 24,457 | 25,147 | 21,749 | 21,935 | 133,332 | 34,592 | 18,127 | 7,971 | 2,506 | 289,816 |
| Pasar Rebo | 17,273 | 17,712 | 15,585 | 15,032 | 89,757 | 23,259 | 12,517 | 4,097 | 1,149 | 196,381 |
| Penjaringan | 23,682 | 24,065 | 21,419 | 22,051 | 136,324 | 32,828 | 19,765 | 8,456 | 2,969 | 291,559 |
| Pesanggrahan | 19,137 | 19,482 | 17,658 | 17,233 | 102,305 | 25,700 | 14,673 | 6,146 | 1,681 | 224,015 |
| Pulo Gadung | 22,129 | 23,079 | 20,725 | 20,841 | 123,240 | 36,079 | 18,559 | 9,328 | 3,555 | 277,535 |
| Sawah Besar | 8,320 | 8,913 | 8,548 | 8,923 | 52,275 | 15,612 | 10,096 | 4,836 | 2,147 | 119,670 |
| Senen | 8,386 | 9,036 | 8,667 | 8,923 | 49,616 | 14,585 | 8,708 | 3,733 | 1,501 | 113,155 |
| Setiabudi | 8,366 | 8,909 | 8,023 | 8,325 | 48,588 | 14,227 | 7,735 | 3,316 | 1,293 | 108,782 |
| Taman Sari | 8,603 | 8,639 | 7,977 | 9,284 | 54,698 | 15,672 | 10,469 | 5,051 | 2,398 | 122,791 |
| Tambora | 20,131 | 20,846 | 18,267 | 20,299 | 120,254 | 30,732 | 17,707 | 7,592 | 2,960 | 258,788 |
| Tanah Abang | 12,550 | 13,324 | 12,495 | 12,680 | 68,930 | 20,035 | 11,010 | 4,670 | 1,856 | 157,550 |
| Tanjung Priok | 32,161 | 32,505 | 28,097 | 29,623 | 176,861 | 43,971 | 23,948 | 9,504 | 2,866 | 379,536 |
| Tebet | 17,554 | 18,059 | 17,090 | 17,625 | 97,205 | 29,860 | 16,406 | 7,488 | 3,098 | 224,385 |
| Total (Jakarta Province) | 819,426 | 832,973 | 742,373 | 759,096 | 4,464,335 | 1,158,241 | 633,406 | 262,111 | 88,341 | 9,760,302 |

## **Mathematical Models**

### 3.1 Incidence Model

The incidence of primary, secondary, tertiary and quaternary infections within each age-group *j* are given by the following equations (Equations 1.1-1.4).

$$I_{1}(j)=\int_{a_{j}}^{a_{j+1}} 4\lambda\left(e^{-\lambda a}\right)^{4}da$$

[1.1]

$$I_{2}(j)=4\int_{a_{j}}^{a_{j+1}} 3\lambda(1-e^{-\lambda a})\left(e^{-\lambda a}\right)^{3}da$$

[1.2]

$I_{3}(j)=4\int_{a_{j}}^{a_{j+1}} 3\lambda(1-e^{-\lambda a})^{2}\left(e^{-\lambda a}\right)^{2}da$

[1.3]

$$I_{4}(j)=4\int_{a_{j}}^{a_{j+1}} \lambda\left(1-e^{-\lambda a}\right)^{3}e^{-\lambda a}da$$

[1.4]

The average annual incidence of dengue disease per person in age-group *j*, *D(j),* is then calculated as the weighted sum of primary and secondary infections (Equation 2):

$$D(j)=\frac{\rho}{w(j)}(I_{2}(j)+\gamma_{1}(I_{1}(j))+\mathrm{B}w(j))$$

[2]

Here *λ* is the age- and time-constant force of infection of each dengue serotype, *a_j_* and *a_j+1_* are the lower and upper bounds of age-group *j*, respectively. *ρ* is the probability that a secondary infection results in a detectable dengue case (baseline reporting rate), *w(j)* is the width of age-group *j*, *γ_1_* is the probability that a primary infection is detected relative to a secondary infection, and *B* is the probability of reporting non-dengue illnesses as dengue due to misdiagnosis (baseline non-dengue reporting). The total force of infection is then calculated as four times the serotype-specific force of infection (i.e. *4λ*). All parameters were given uniform priors (range 0-1).

### 3.2 Likelihood function

The expected number of cases per year in age-group j, C_j_, is given by:

$$C_{j}=D(j)n(j)$$

[3]

Where *D(j)* is the expected disease incidence in age-group *j*, and *n(j)* is the population size of age-group *j*.

We assumed that the total number of cases across all ages, N, was Poisson distributed (Equation 4):

$$P(\mu=N)=\frac{\mu^{N}e^{-\mu}}{N!}$$

[4]

Where μ is the total expected number of cases across all age-groups (i.e. ΣC_j_). We assumed that the number of cases reported in each age-group were multinomially distributed and so the full log-likelihood is given by:

$\ln L=\sum_{j} \left[ \ln(p_{j})y_{j} \right]+N\ln(\mu)-\mu-\ln(N!)$

[5]

where *p_j_* is the expected proportion of cases in one age-group relative to the total number of cases across all age-groups (i.e*. C_j_ / Σ_j_C_j_*) and *y_j_* is the observed number of cases in age-group *j*.

In Model 2, where we estimate age-constant and time-varying (piecewise-constant) force of infection, we assume a multinomial age-distribution for each year worth of data, so the full log-likelihood is given by:

$\ln L=\sum_{t} \left\{ \sum_{j} \left[ \ln(p_{jt})y_{jt} \right]+N_{t}\ln(\mu_{t})-\mu_{t}-\ln(N_{t}!) \right\}$

where *p_jt_* denotes the expected proportion of cases in age-group *j* in year *t, y_jt_* denotes the observed number of cases in age-group *j* in year *t*, *N_t_* denotes the total number of cases across all ages in year *t* and *μ_t_* is the total expected number of cases across all age-groups in year *t.*

### 3.3 Seropositive proportion of the population

The proportion of the population seropositive to at least one dengue serotype by age is determined by the force of infection, *λ*. Given a total force of infection of *4λ* (assuming equal transmissibility across serotypes), the expected proportion of the population seropositive to dengue by age *a*, *p(a)*, is defined by the catalytic model as shown in Equation 6.

$$p(a)=1-e^{(-4\lambda a)}$$

[6]

### 3.4 Basic Reproduction Number

Assuming that dengue force of infection was constant in time and equally transmissible across all 4 serotypes, we calculated the basic reproduction number of each serotype, *R_0_*, under two assumptions. Under assumption 1, we assumed that all infections (primary-quaternary) are equally infectious, with no cross-immunity between serotypes, shown in Equation 7:

$$R_{0}=\frac{1}{1-\int_{a_{j}}^{a_{j+1}} f(a)[1-e^{-\lambda a}]da}$$

[7]

Where *f(a)* is the proportion of the population in age-group a, [*1-e^-λa^*] is the proportion of the population seropositive to one serotype at age *a*, and *a_j_* and *a_j+1_* are the lower and upper bounds of age-group *j*.

Under assumption 2, we assume that only primary and secondary infections are infectious and contribute to onward transmission. The basic reproduction number for any one serotype is then given by:

$$R_{_{0}}=\frac{1}{\int_{a_{j}}^{a_{j+1}} f(a)[e^{-\lambda a}+(n-1)(1-e^{-\lambda a})]da}$$

[8]

Where *f(a)* is the proportion of the population in age-group *a, e^-λa^* is the proportion seronegative at age a, n is the number of serotypes in circulation (4 in this case), [*1-e^-λa^*] is the proportion seropositive at age *a,* and *a_j_* and *a_j+1_* are the lower and upper bounds of age-group *j*.

### 3.5 Linear regression for prediction of force of infection

A simple linear regression model was used to determine the relationship between subdistrict force of infection and population density, as follows:

$$Y=\beta_{1}+\beta_{2}X+\varepsilon$$

[9]

Where *Y* is the force of infection (*4λ), X* is the population density, *β_1_* is the intercept, *β_2_* is the slope, and ε is the error term, which was assumed to be Normally distributed.

## **Results**

### Jakarta province: parameter estimates and model fits from yearly and cumulative data

**Table D. Median parameter estimates, 95% credible intervals (CrI) and DIC values obtained from the fit of model variants *S* and *PS* to individual years and cumulative data reported in the period 2008-2017 in Jakarta province. Differences in DIC values (*S* – *PS*) show which model variant provided a better fit to the data (negative values favour model variant *S* and positive values favour *PS*).**

| **Year/Period** | **Model** | ***4λ***  **median and (95% CrI)** | ***ρ***  **median and (95%CrI)** | ***γ_1_***  **median and (95%CrI)** | ***β***  **median and (95%CrI)** | **DIC** | **ΔDIC**  **(*S - PS*)** |
| --- | --- | --- | --- | --- | --- | --- | --- |
| 2008 | $S$ | 0.119 (0.116-0.121) | 0.114 (0.109-0.118) | - | 0.009 (0.008-0.010) | 93659.871 | -2.940 |
|  | $PS$ | 0.118 (0.115-0.121) | 0.113 (0.109-0.118) | 0.004 (0.001-0.014) | 0.009 (0.008-0.010) | 93662.811 |  |
| 2009 | $S$ | 0.120 (0.117-0.123) | 0.115 (0.111-0.119) | - | 0.009 (0.008-0.010) | 93635.849 | -3.046 |
|  | $PS$ | 0.119 (0.117-0.122) | 0.115 (0.111-0.119) | 0.003 (0.001-0.013) | 0.009 (0.008-0.010) | 93638.895 |  |
| 2010 | $S$ | 0.131 (0.128-0.133) | 0.112 (0.109-0.116) | - | 0.010 (0.009-0.011) | 96789.824 | -3.207 |
|  | $PS$ | 0.130 (0.127-0.133) | 0.112 (0.109-0.116) | 0.002 (0.001-0.011) | 0.010 (0.009-0.011) | 96793.031 |  |
| 2011 | $S$ | 0.140 (0.134-0.147) | 0.032 (0.030-0.034) | - | 0.018 (0.015-0.020) | 36869.987 | 21.289 |
|  | $PS$ | 0.118 (0.107-0.129) | 0.031 (0.028-0.033) | 0.182 (0.102-0.277) | 0.016 (0.014-0.019) | 36848.698 |  |
| 2012 | $S$ | 0.147 (0.140-0.154) | 0.033 (0.031-0.035) | - | 0.020 (0.018-0.023) | 42197.232 | 17.356 |
|  | $PS$ | 0.125 (0.114-0.137) | 0.031 (0.029-0.034) | 0.170 (0.088-0.268) | 0.020 (0.017-0.022) | 42179.876 |  |
| 2013 | $S$ | 0.135 (0.131-0.139) | 0.063 (0.060-0.066) | - | 0.013 (0.012-0.015) | 67245.316 | -0.133 |
|  | $PS$ | 0.131 (0.125-0.136) | 0.062 (0.059-0.065) | 0.029 (0.002-0.072) | 0.013 (0.012-0.015) | 67245.449 |  |
| 2014 | $S$ | 0.138 (0.133-0.142) | 0.054 (0.051-0.057) | - | 0.016 (0.015-0.018) | 64541.894 | 17.735 |
|  | $PS$ | 0.124 (0.116-0.131) | 0.052 (0.049-0.055) | 0.114 (0.061-0.173) | 0.015 (0.014-0.017) | 64524.159 |  |
| 2015 | $S$ | 0.141 (0.135-0.148) | 0.030 (0.028-0.032) | - | 0.020 (0.018-0.023) | 41955.472 | 34.45 |
|  | $PS$ | 0.115 (0.105-0.126) | 0.028 (0.026-0.031) | 0.228 (0.144-0.330) | 0.020 (0.017-0.023) | 41921.022 |  |
| 2016 | $S$ | 0.136 (0.134-0.138) | 0.179 (0.176-0.182) | - | 0.005 (0.005-0.005) | 137876.585 | -5.517 |
|  | $PS$ | 0.135 (0.134-0.137) | 0.179 (0.176-0.182) | 0.001 (0.001-0.004) | 0.005 (0.005-0.005) | 137882.102 |  |
| 2017 | $S$ | 0.178 (0.171-0.185) | 0.026 (0.025-0.028) | - | 0.013 (0.012-0.014) | 29243.400 | 14.061 |
|  | $PS$ | 0.159 (0.148-0.171) | 0.024 (0.022-0.026) | 0.158 (0.076-0.255) | 0.013 (0.012-0.015) | 29229.339 |  |
| 2008-2017 | $S$ | 0.130 (0.129-0.131) | 0.077 (0.076-0.078) | - | 0.010 (0.010-0.010) | 705596.266 | -2.548 |
|  | $PS$ | 0.130 (0.129-0.131) | 0.077 (0.076-0.077) | 0.003 (0.001-0.010) | 0.010 (0.010-0.010) | 705598.814 |  |

**Figure S1. Fit of model variants *S* (orange line and ribbon indicating median and 95% credible intervals) and *PS* (green line and ribbon indicating median and 95% credible intervals) to yearly and cumulative (2008-2017) age-stratified DHF incidence rates reported in Jakarta province (black points and bars, indicating mean and 95% Binomial confidence intervals).**


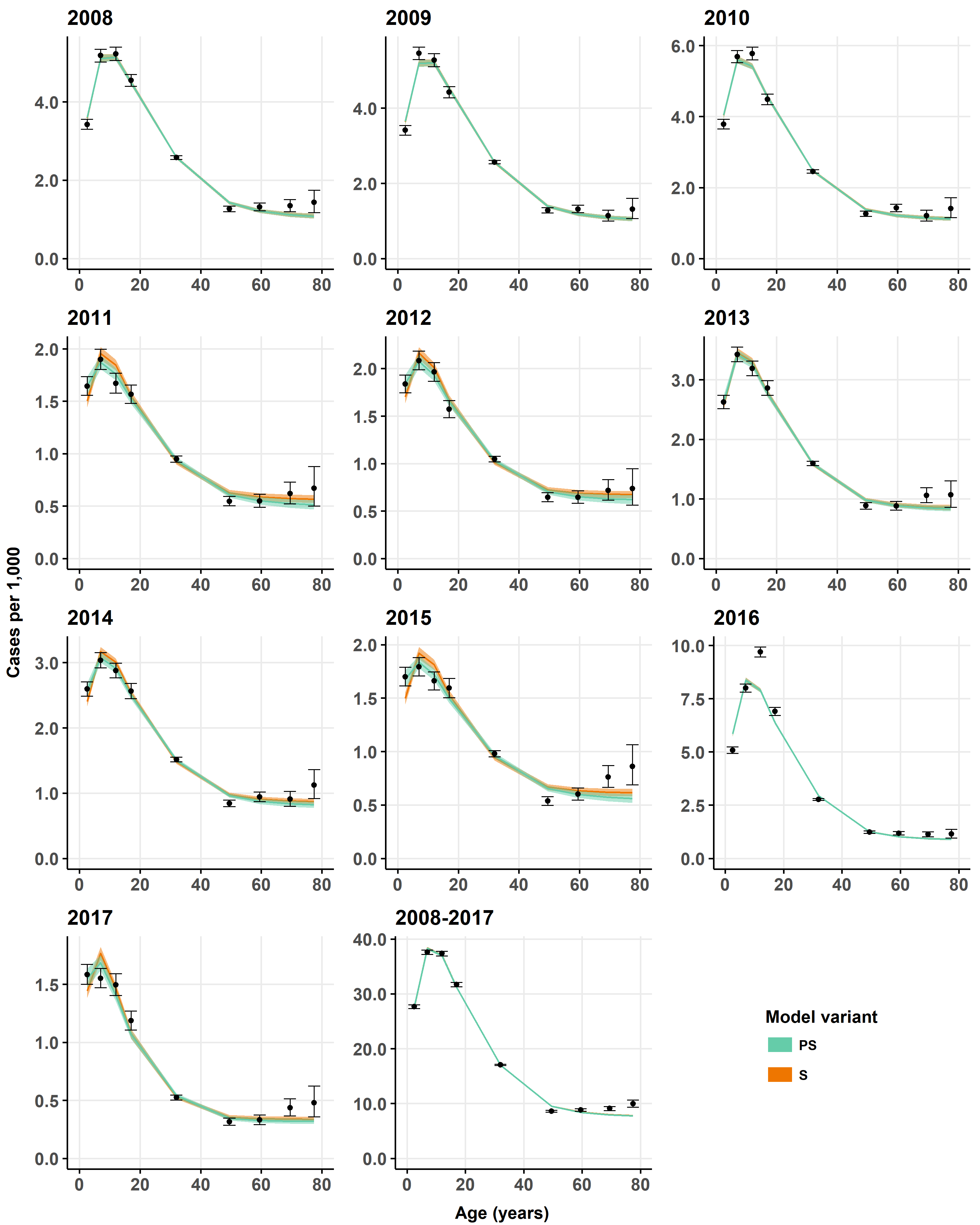


### Jakarta subdistricts: parameter estimates and model fits from cumulative data

**Figure S2. Median and 95% credible interval of parameter estimates, log-likelihood (LnL) and DIC values obtained from the fit of model variants *S* (orange) and *PS* (green) to cumulative age-stratified DHF data (2008-2017) reported in Jakarta’s subdistricts. Subdistrict parameter estimates from yearly data are given in Supplementary file S3.**


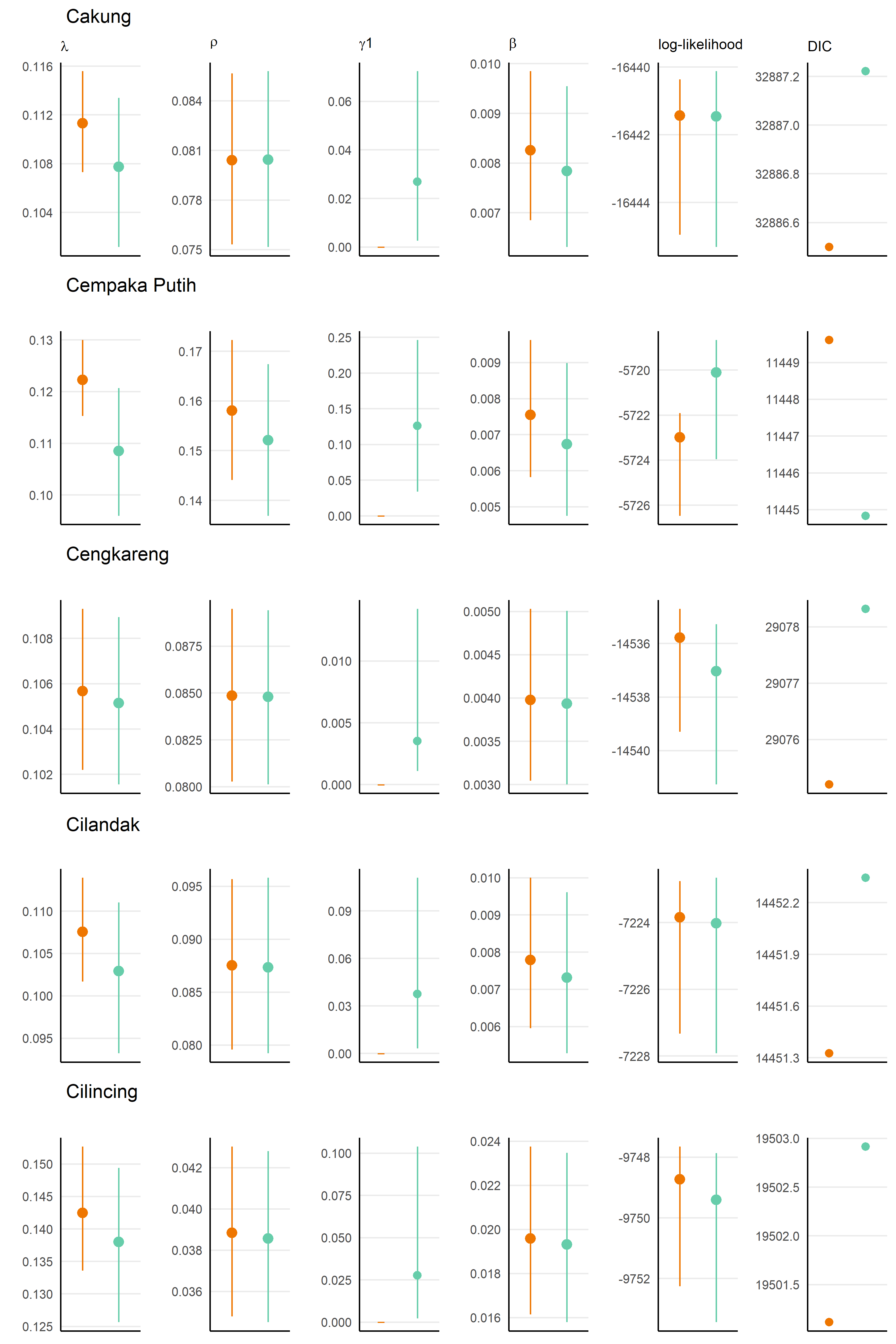


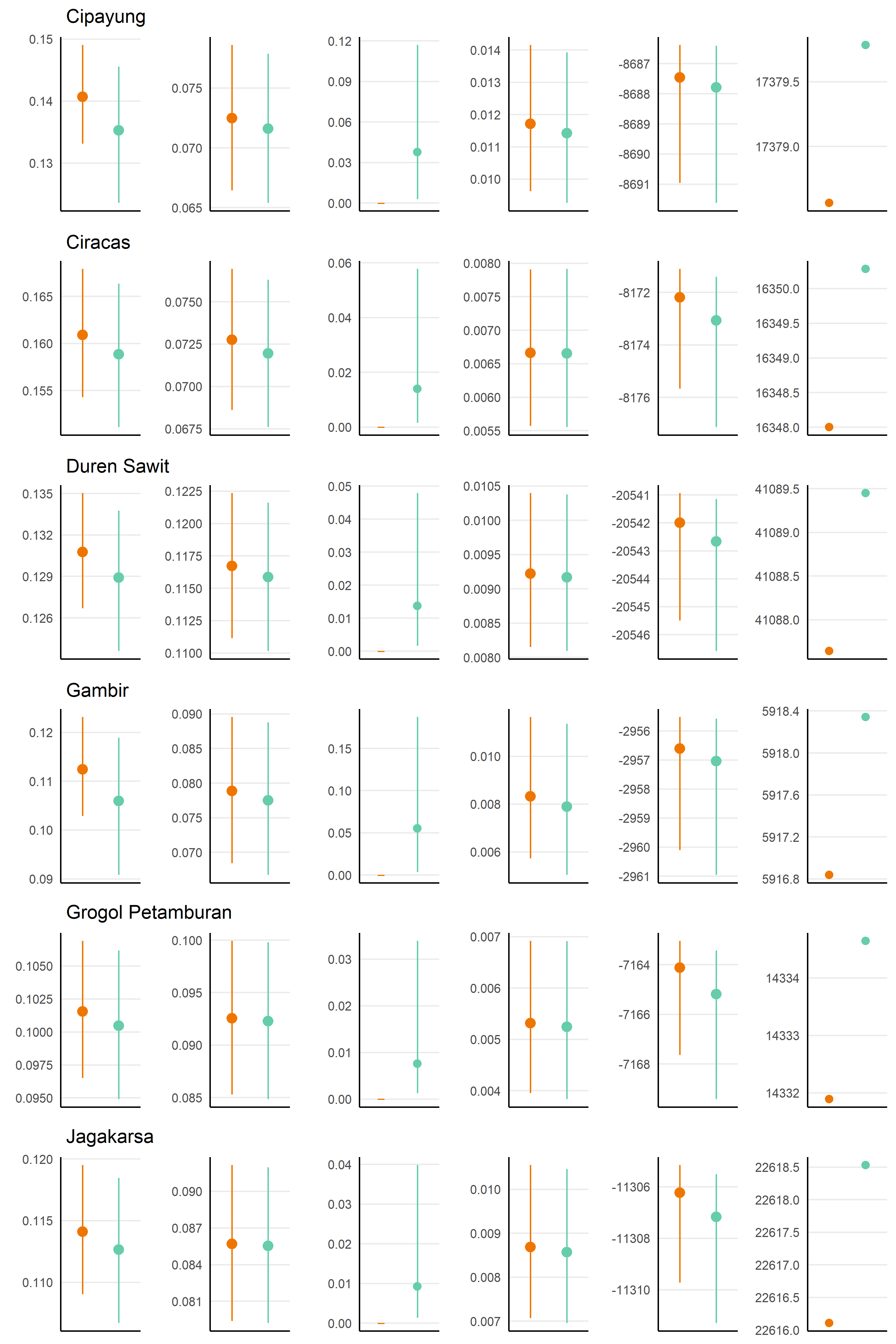


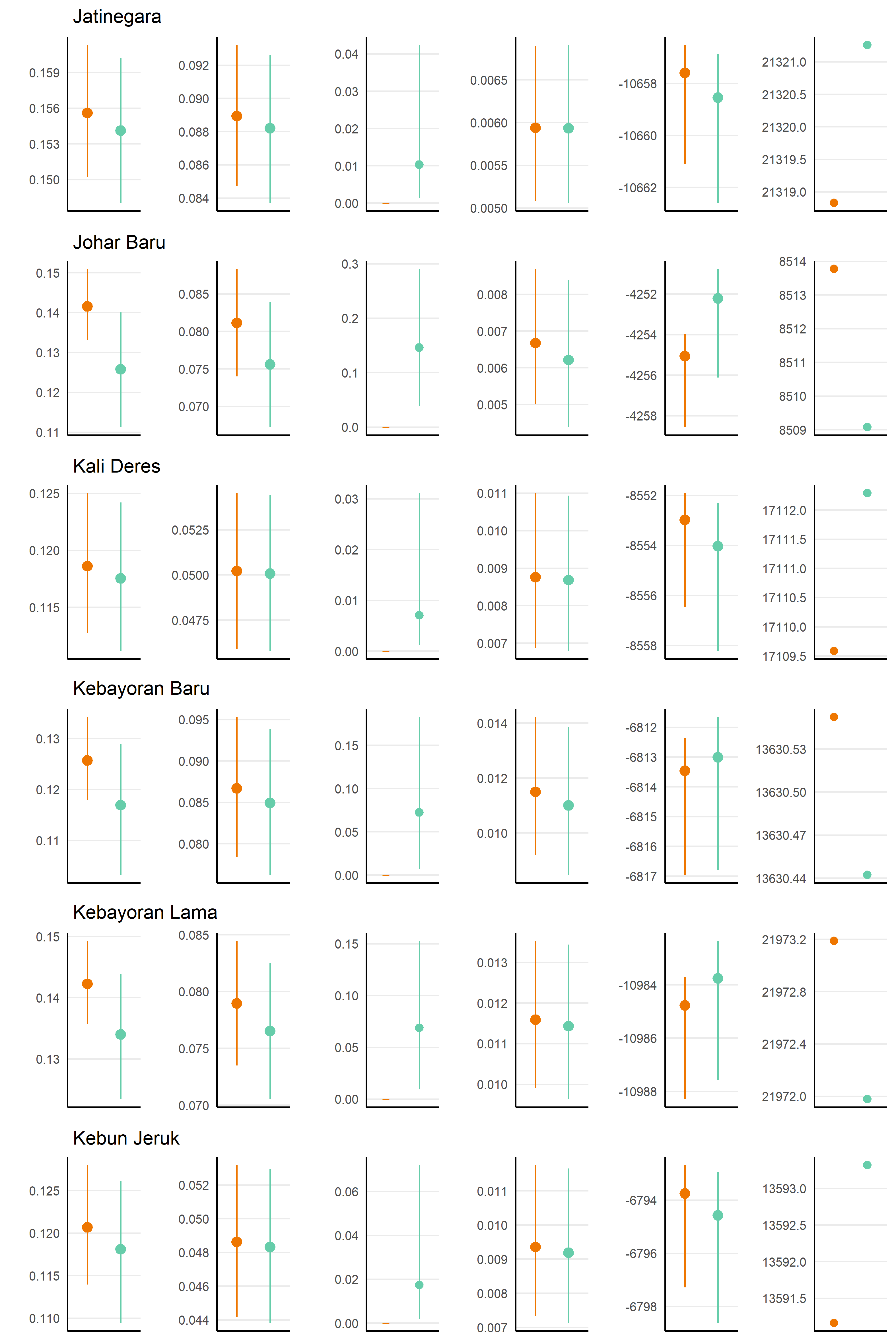


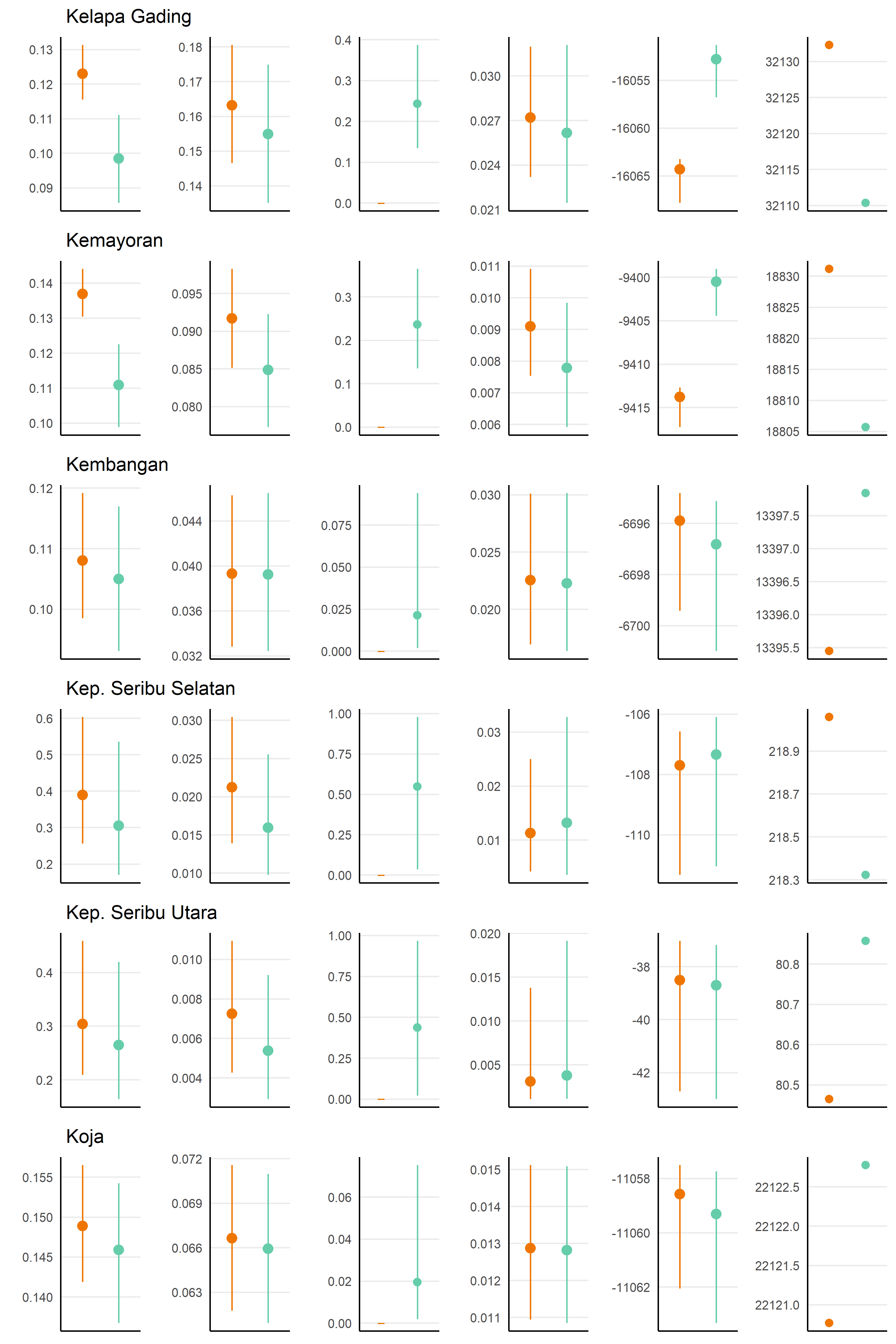


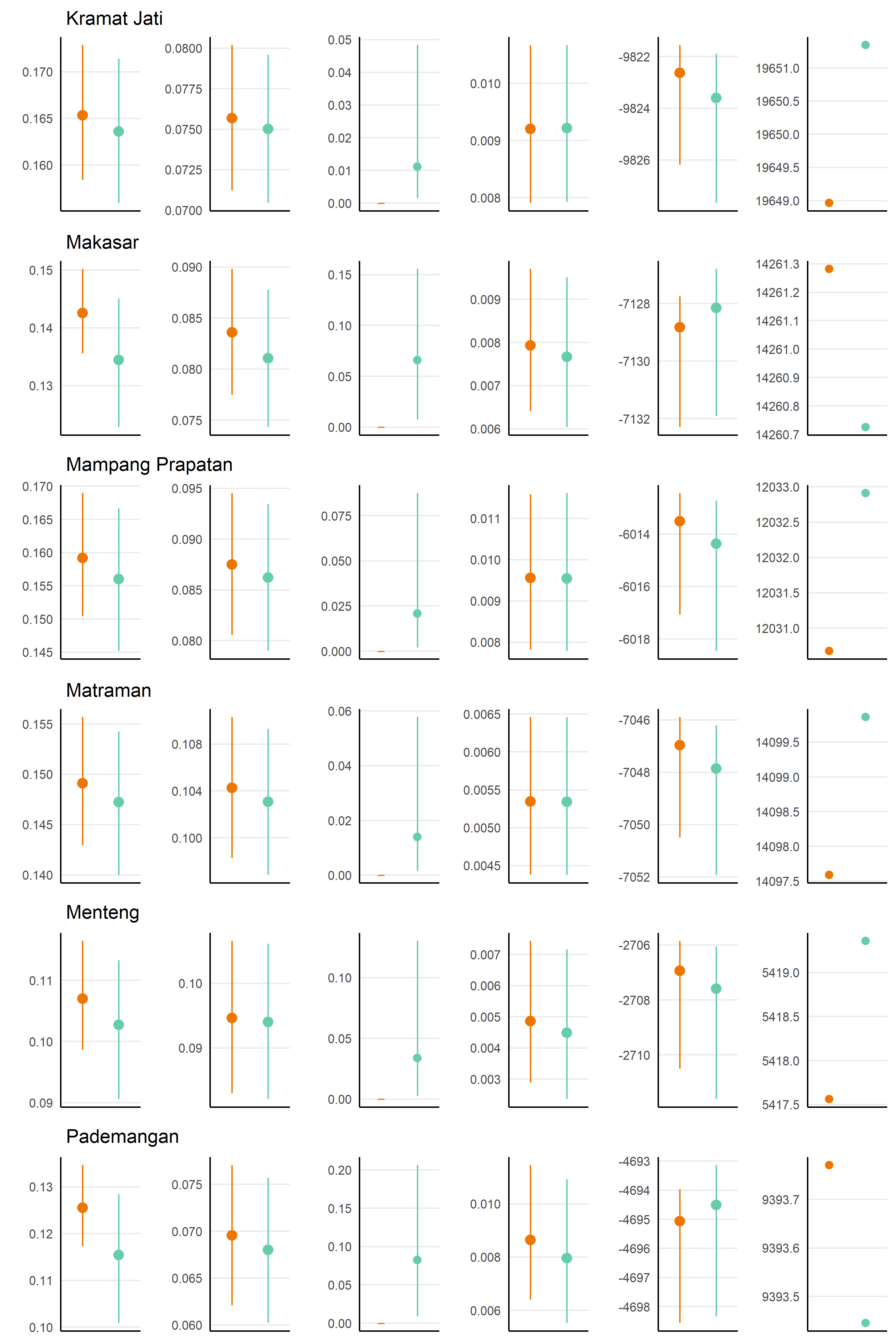


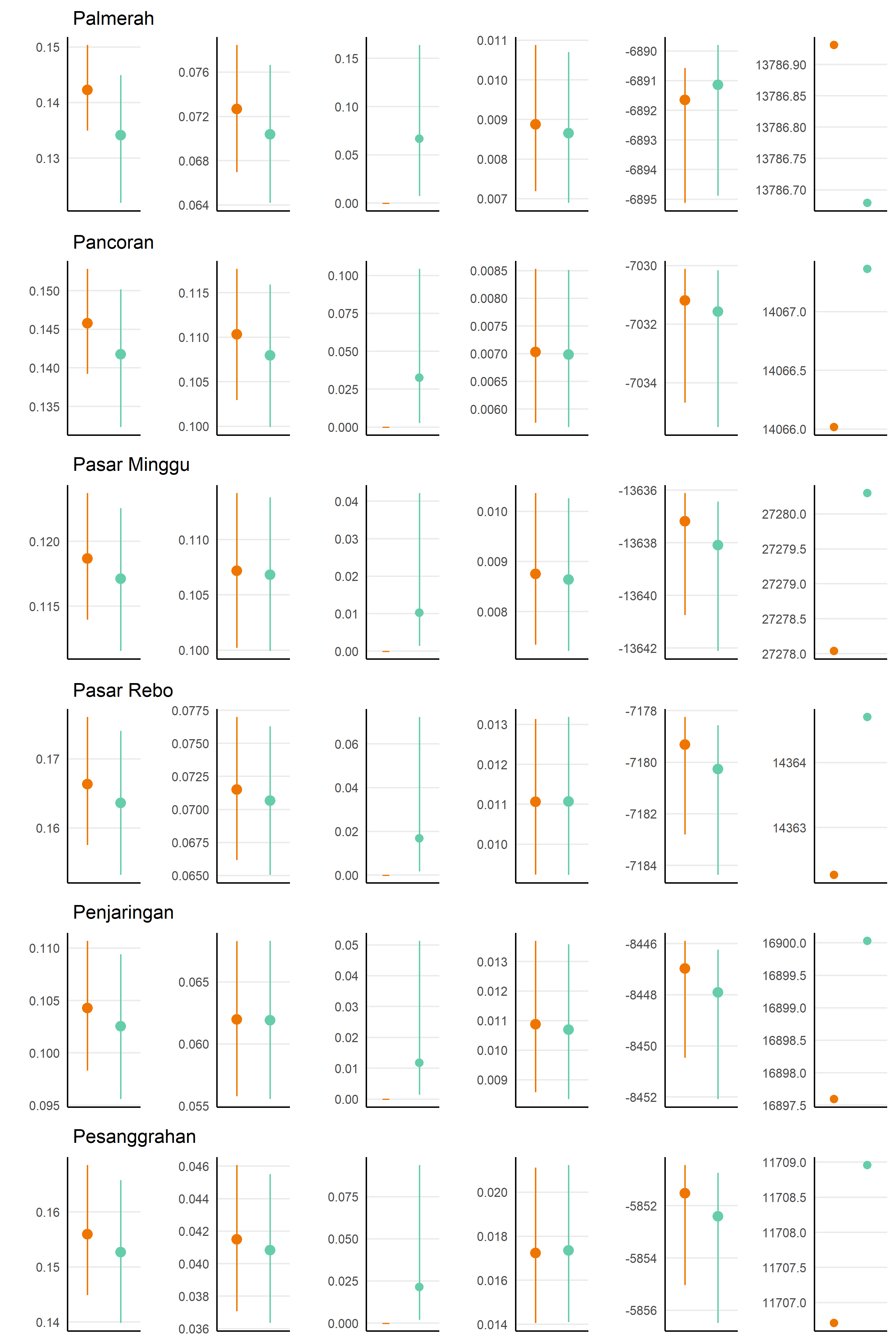


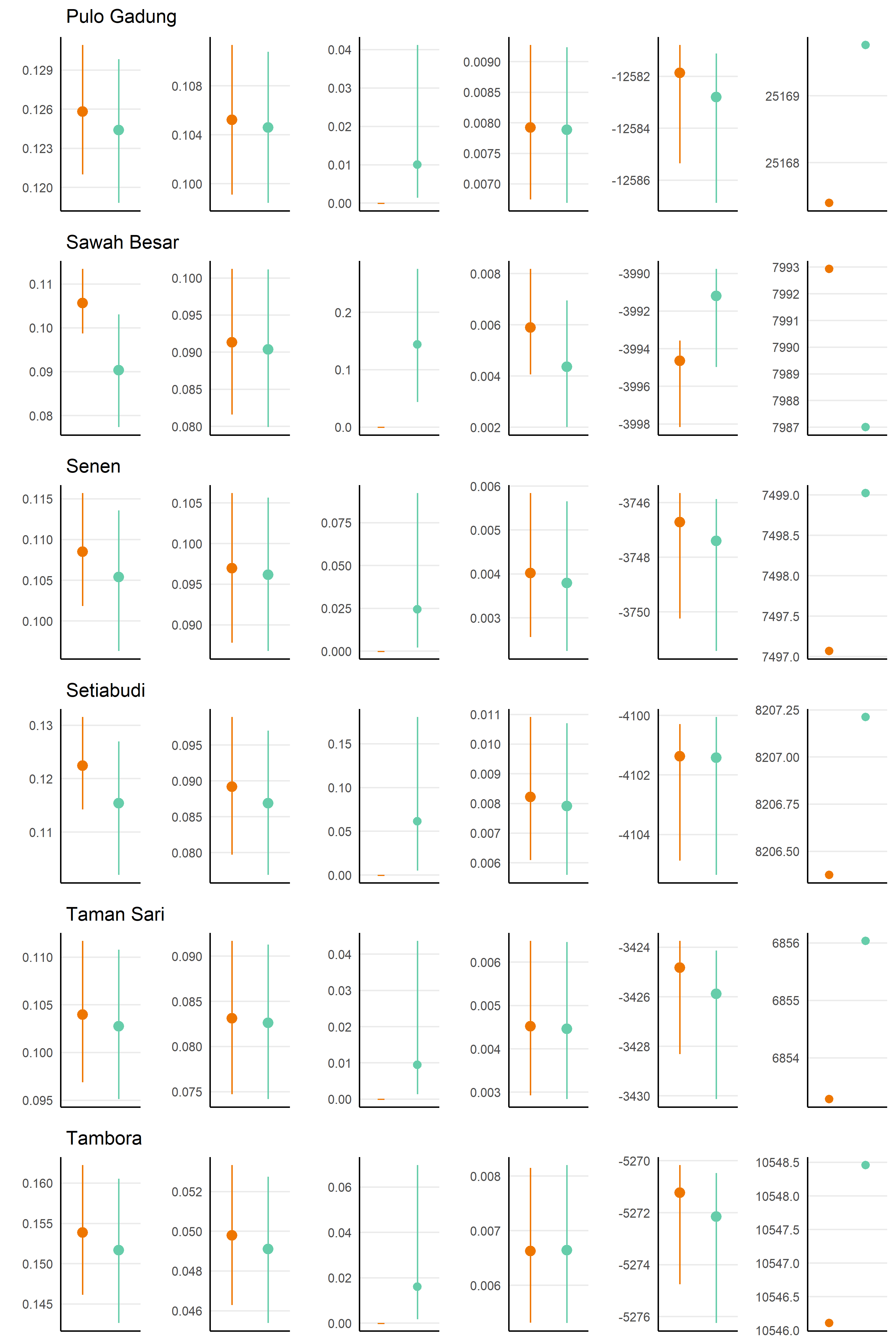


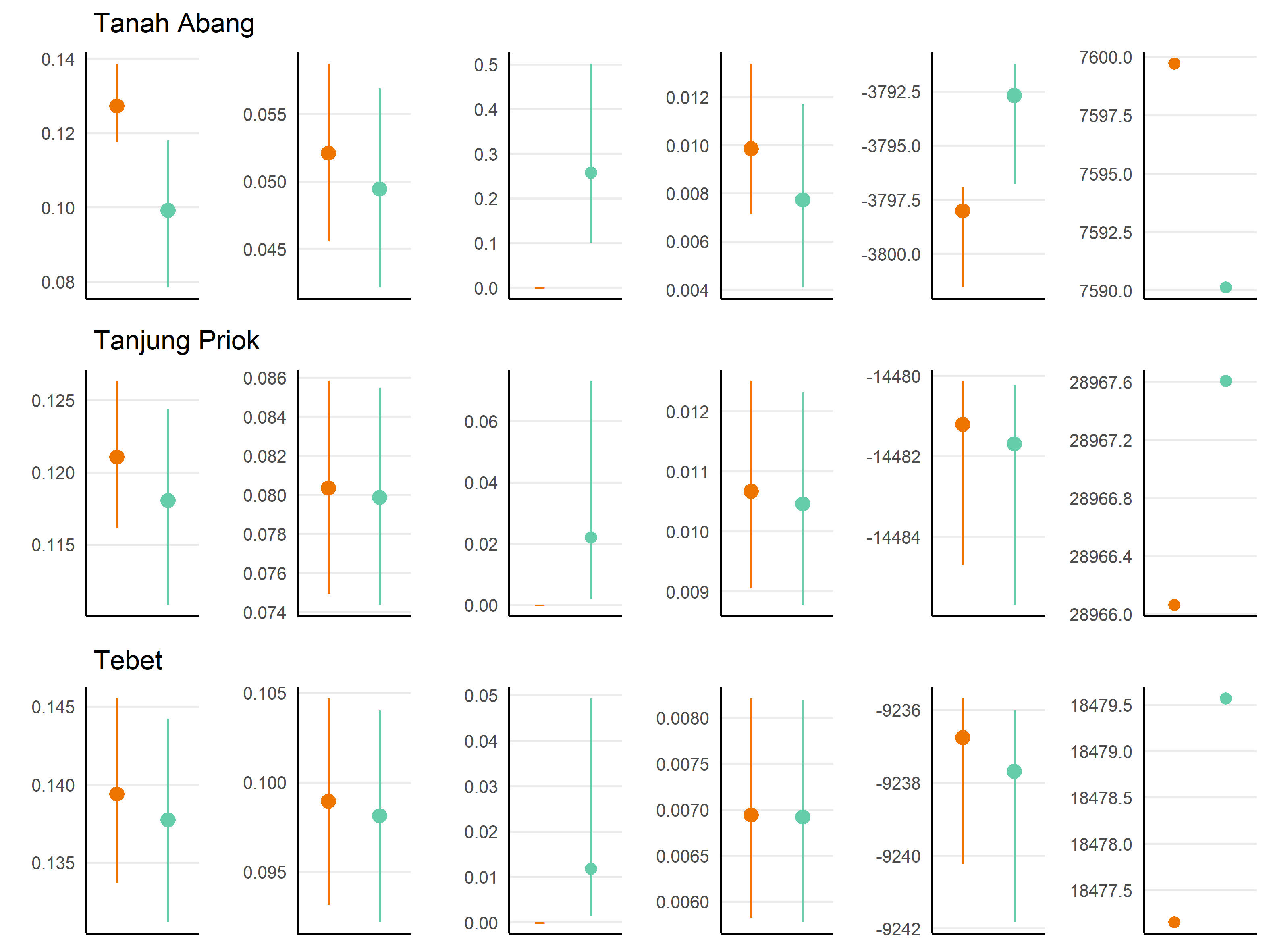


**Figure S3. Fit of model variants *S* (orange line and ribbon, indicating median and 95% credible intervals) and *PS* (green line and ribbon, indicating median and 95% credible intervals) to cumulative (2008-2017) DHF incidence rates by age (black points and bars, indicating mean and 95% Binomial confidence intervals) reported in Jakarta’s subdistricts.**


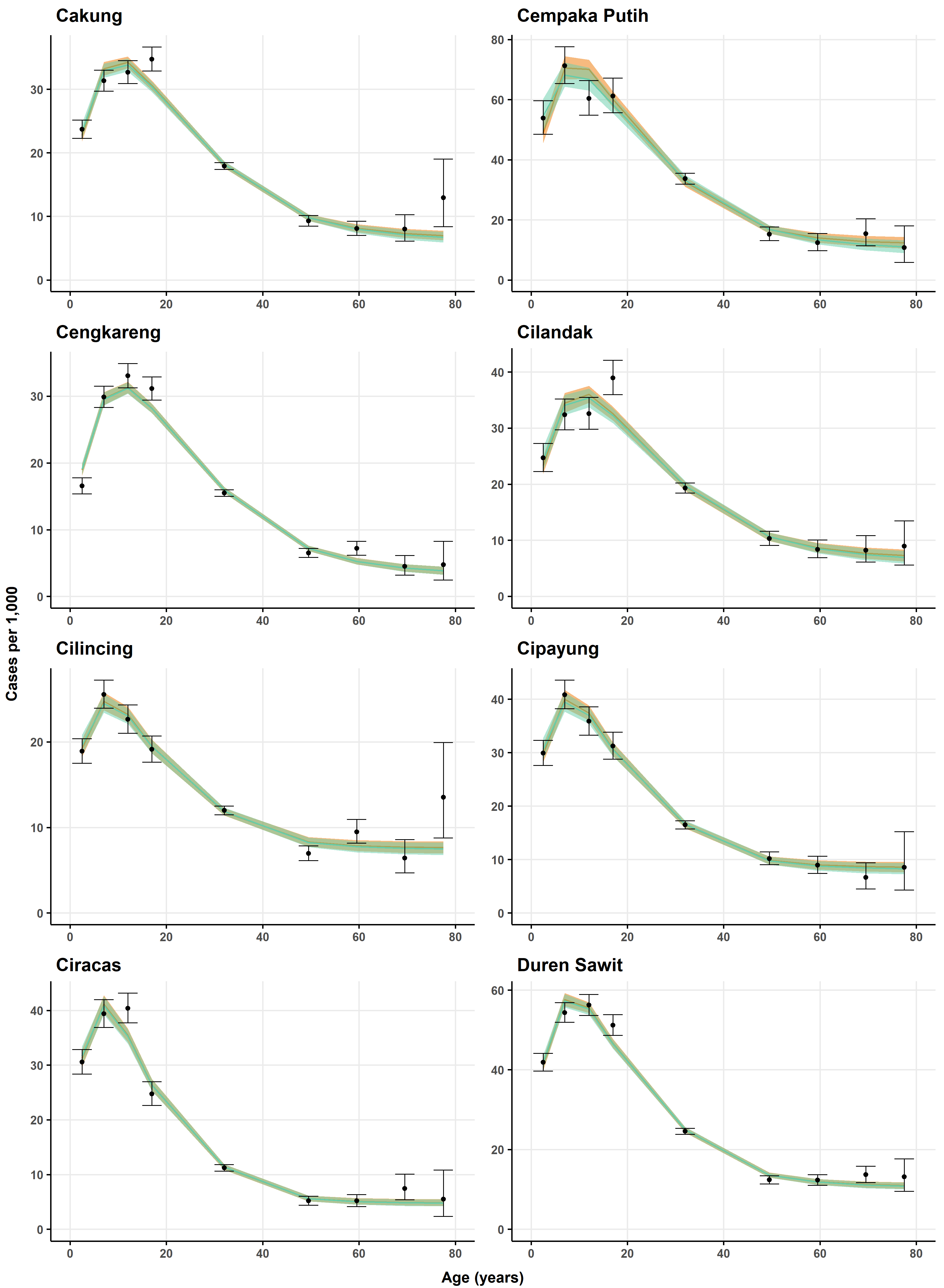


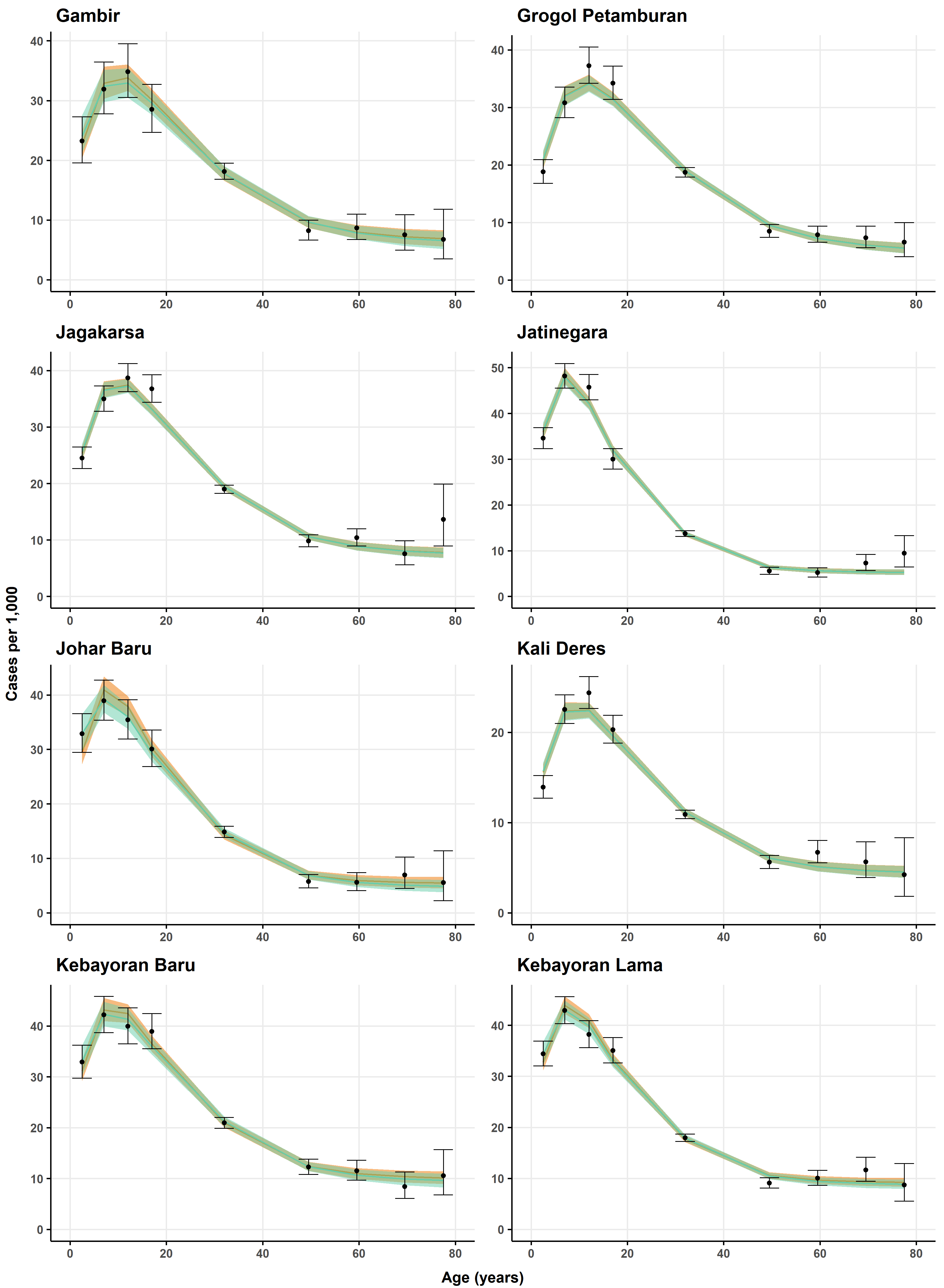


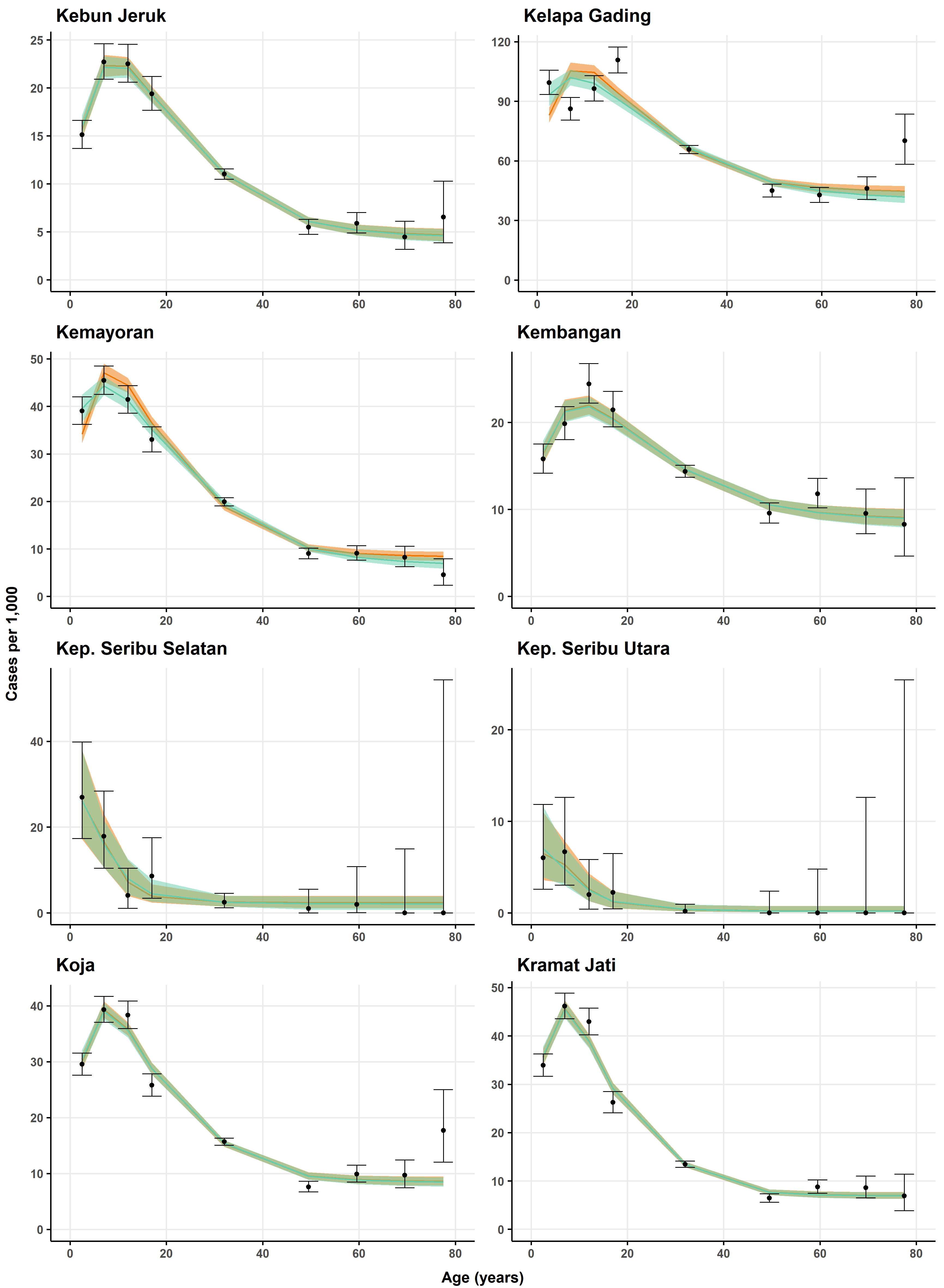


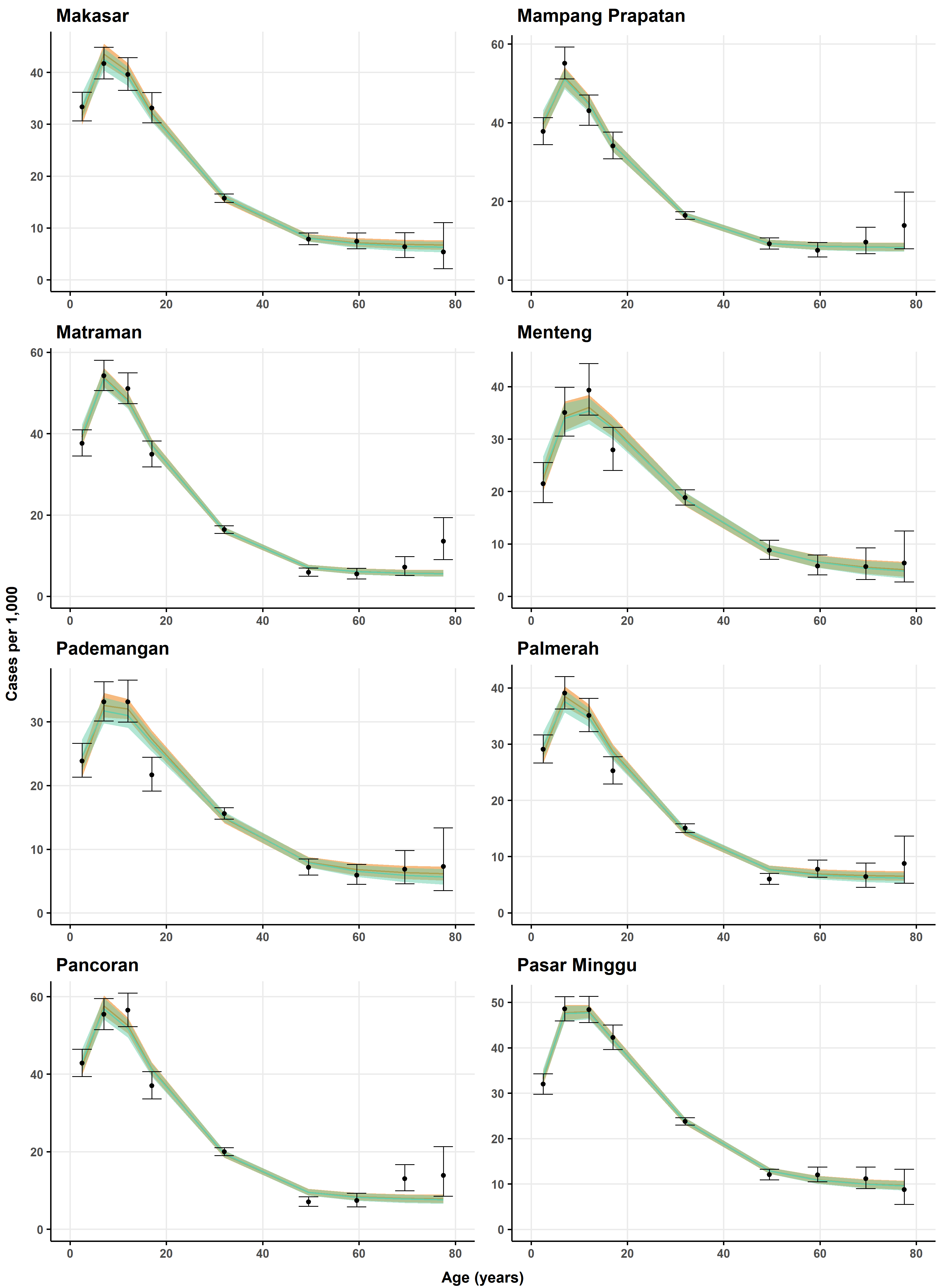


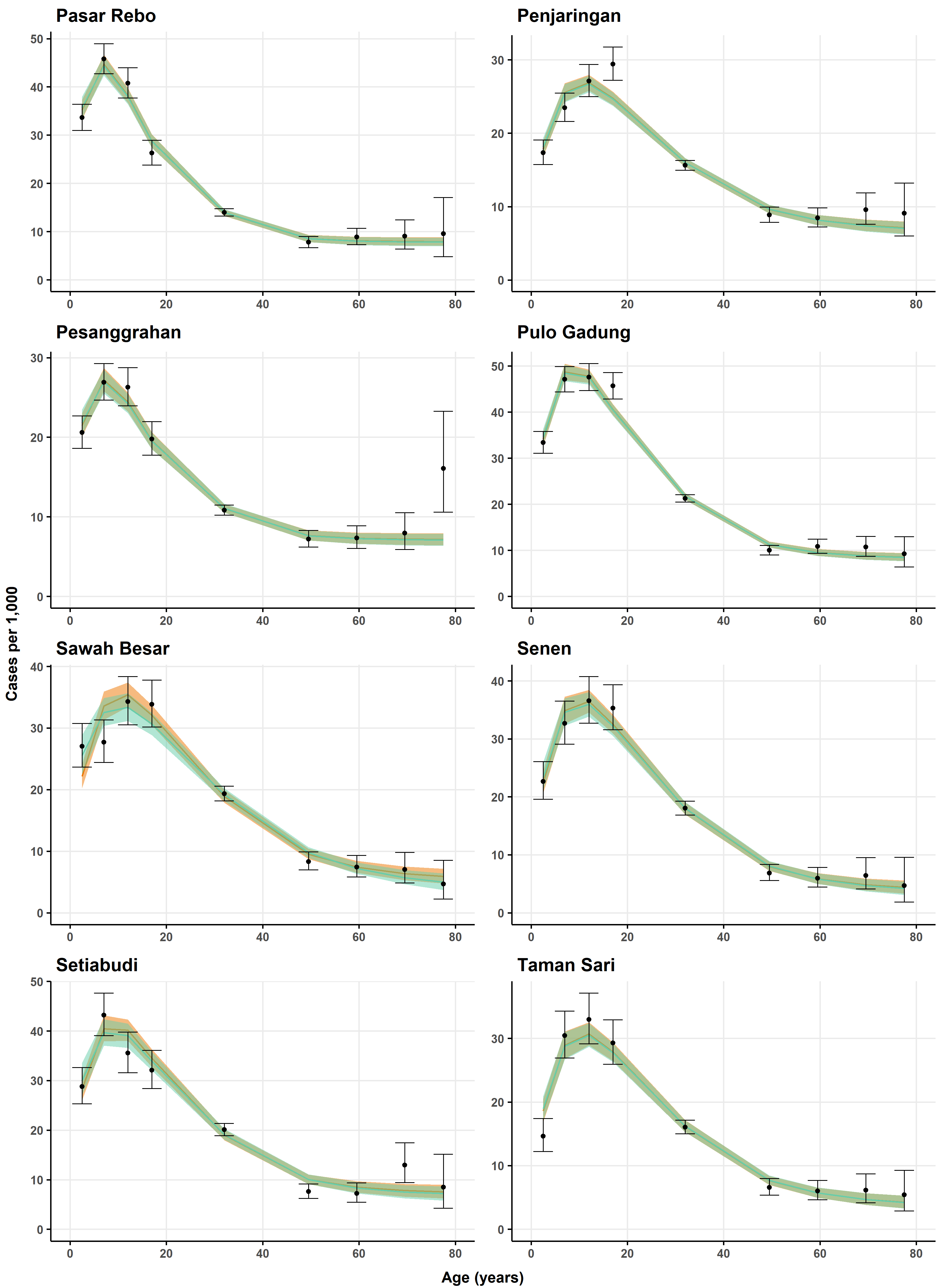

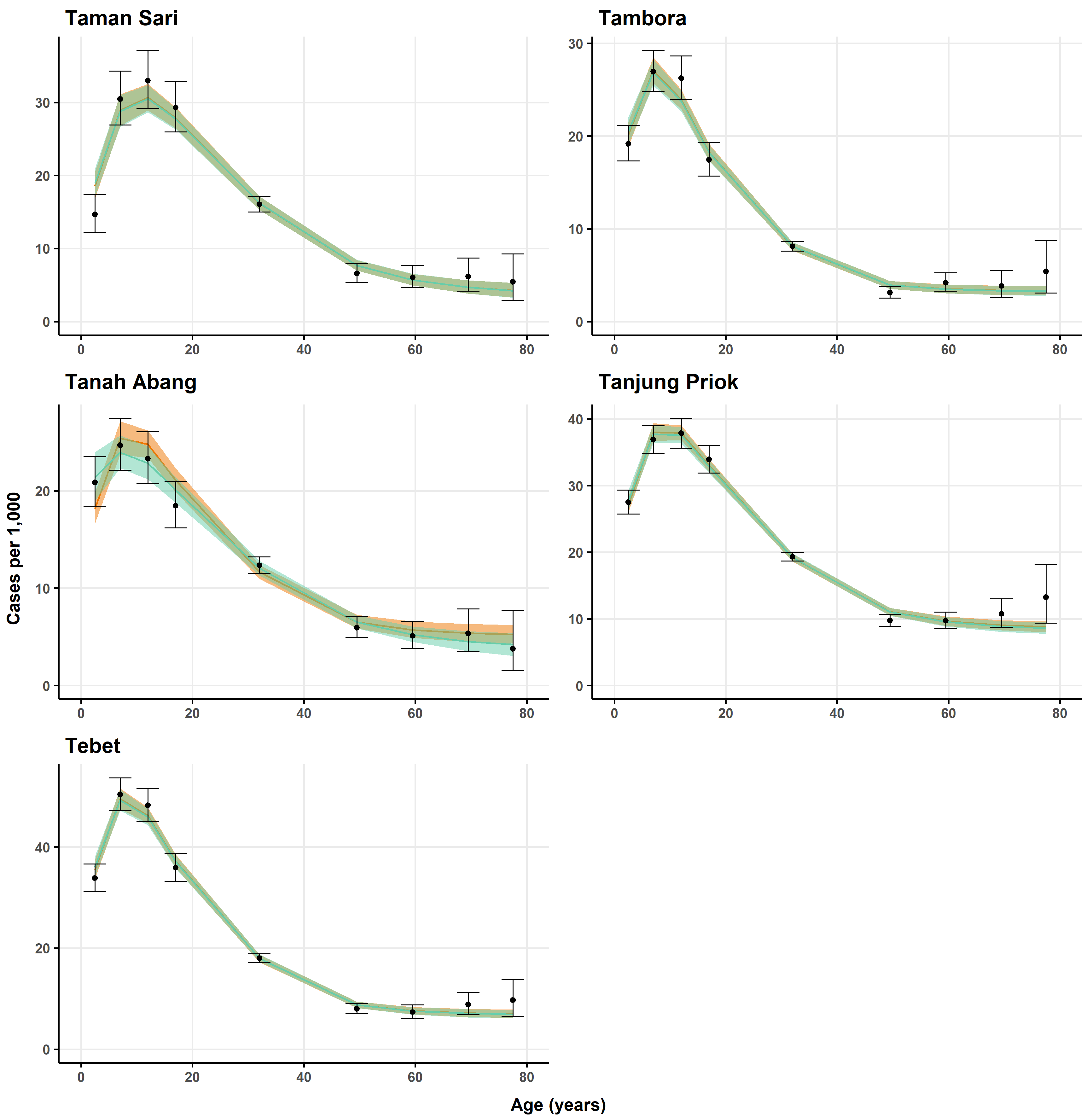


**Table E. Estimated total force of infection, basic reproduction numbers, the proportion of seropositive children at 9 years of age obtained by fitting model 1 variant PS to cumulative (2008-2017) DHF data reported in Jakarta’s subdistricts, and subdistrict population density. The basic reproduction number (R0) was estimated under 2 assumptions: under assumption 1 we assume that primary to quaternary infections are equally infectious; under assumption 2 we assume that only primary and secondary infections are infectious and thus contribute to onward transmission. The subdistrict population density estimates are reported online (2).**

| **Subdistrict** | **Total force of infection, 4λ,**  **(median and 95%CrI)** | **Proportion of 9 year olds seropositive (median and 95%CrI)** | **R0 [assumption 1]**  **(median and 95%CrI)** | **R0 [assumption 2]**  **(median and 95%CrI)** | **Population density per km^2^** |
| --- | --- | --- | --- | --- | --- |
| Cakung | 0.108 (0.101-0.113) | 0.621 (0.598-0.640) | 1.961 (1.892-2.020) | 3.112 (2.940- 3.261) | 12,362 |
| Cempaka Putih | 0.108 (0.096-0.121) | 0.623 (0.578-0.662) | 2.099 (1.950-2.249) | 3.490 (3.105- 3.877) | 18,223 |
| Cengkareng | 0.105 (0.102-0.109) | 0.612 (0.599-0.625) | 1.948 (1.910-1.989) | 3.097 (2.999- 3.200) | 19,589 |
| Cilandak | 0.103 (0.093-0.111) | 0.604 (0.568-0.632) | 2.004 (1.893-2.099) | 3.243 (2.958- 3.488) | 10,685 |
| Cilincing | 0.138 (0.126-0.149) | 0.711 (0.677-0.739) | 2.265 (2.131-2.392) | 3.866 (3.535- 4.176) | 8,923 |
| Cipayung | 0.135 (0.124-0.146) | 0.704 (0.671-0.730) | 2.286 (2.154-2.405) | 3.919 (3.593- 4.208) | 8,215 |
| Ciracas | 0.159 (0.151-0.166) | 0.761 (0.743-0.776) | 2.600 (2.506-2.692) | 4.702 (4.474- 4.924) | 15,137 |
| Duren Sawit | 0.129 (0.124-0.134) | 0.687 (0.671-0.700) | 2.288 (2.224-2.346) | 3.944 (3.785- 4.090) | 17,637 |
| Gambir | 0.106 (0.091-0.119) | 0.614 (0.558-0.657) | 2.122 (1.933-2.291) | 3.576 (3.078- 4.021) | 10,438 |
| Grogol Petamburan | 0.100 (0.095-0.106) | 0.595 (0.574-0.615) | 2.019 (1.952-2.089) | 3.307 (3.131- 3.490) | 20,539 |
| Jagakarsa | 0.113 (0.107-0.118) | 0.637 (0.617-0.656) | 2.046 (1.981-2.110) | 3.325 (3.162- 3.485) | 12,304 |
| Jatinegara | 0.154 (0.148-0.160) | 0.75 (0.736-0.764) | 2.610 (2.533-2.689) | 4.731 (4.544- 4.920) | 25,865 |
| Johar Baru | 0.126 (0.111-0.140) | 0.678 (0.633-0.716) | 2.238 (2.070-2.410) | 3.821 (3.395- 4.252) | 49,162 |
| Kali Deres | 0.118 (0.111-0.124) | 0.653 (0.632-0.673) | 2.061 (1.993-2.134) | 3.383 (3.209- 3.567) | 13,881 |
| Kebayoran Baru | 0.117 (0.103-0.129) | 0.651 (0.605-0.687) | 2.202 (2.035-2.353) | 3.760 (3.329- 4.147) | 11,163 |
| Kebayoran Lama | 0.134 (0.123-0.144) | 0.701 (0.671-0.726) | 2.366 (2.237-2.490) | 4.169 (3.841- 4.480) | 15,157 |
| Kebun Jeruk | 0.118 (0.109-0.126) | 0.655 (0.627-0.679) | 2.154 (2.054-2.249) | 3.632 (3.374- 3.874) | 19,434 |
| Kelapa Gading | 0.098 (0.086-0.111) | 0.588 (0.537-0.632) | 2.024 (1.867-2.185) | 3.327 (2.912- 3.752) | 9,607 |
| Kemayoran | 0.111 (0.099-0.123) | 0.631 (0.589-0.668) | 2.085 (1.948-2.223) | 3.432 (3.083- 3.781) | 30,198 |
| Kembangan | 0.105 (0.093-0.117) | 0.611 (0.567-0.651) | 1.984 (1.855-2.120) | 3.192 (2.859- 3.539) | 10,814 |
| Koja | 0.146 (0.137-0.154) | 0.731 (0.708-0.750) | 2.394 (2.289-2.490) | 4.185 (3.930- 4.420) | 25,195 |
| Kramat Jati | 0.164 (0.156-0.171) | 0.771 (0.754-0.786) | 2.667 (2.572-2.764) | 4.843 (4.618- 5.073) | 20,671 |
| Makasar | 0.134 (0.123-0.145) | 0.702 (0.669-0.729) | 2.323 (2.187-2.450) | 4.028 (3.689- 4.342) | 8,751 |
| Mampang Prapatan | 0.156 (0.145-0.167) | 0.754 (0.729-0.777) | 2.607 (2.472-2.743) | 4.759 (4.423- 5.090) | 17,927 |
| Matraman | 0.147 (0.140-0.154) | 0.734 (0.716-0.750) | 2.559 (2.466-2.650) | 4.625 (4.396- 4.848) | 30,203 |
| Menteng | 0.103 (0.091-0.113) | 0.603 (0.557-0.639) | 2.045 (1.900-2.175) | 3.351 (2.978- 3.687) | 10,506 |
| Pademangan | 0.115 (0.101-0.128) | 0.646 (0.597-0.685) | 2.117 (1.952-2.268) | 3.533 (3.108- 3.919) | 12,239 |
| Palmerah | 0.134 (0.122-0.145) | 0.701 (0.666-0.729) | 2.347 (2.200-2.480) | 4.112 (3.741- 4.444) | 26,998 |
| Pancoran | 0.142 (0.132-0.150) | 0.721 (0.696-0.741) | 2.446 (2.330-2.551) | 4.352 (4.063- 4.611) | 16,703 |
| Pasar Minggu | 0.117 (0.112-0.123) | 0.651 (0.634-0.668) | 2.131 (2.068-2.195) | 3.559 (3.397- 3.719) | 13,345 |
| Pasar Rebo | 0.164 (0.153-0.174) | 0.771 (0.748-0.791) | 2.644 (2.517-2.773) | 4.797 (4.493- 5.103) | 14,599 |
| Penjaringan | 0.103 (0.096-0.109) | 0.603 (0.577-0.626) | 1.984 (1.906-2.063) | 3.196 (2.994- 3.400) | 8,461 |
| Pesanggrahan | 0.153 (0.140-0.166) | 0.747 (0.716-0.775) | 2.542 (2.386-2.704) | 4.569 (4.185- 4.962) | 15,678 |
| Pulo Gadung | 0.124 (0.119-0.130) | 0.674 (0.657-0.689) | 2.265 (2.197-2.332) | 3.903 (3.730- 4.072) | 17,506 |
| Sawah Besar | 0.090 (0.077-0.103) | 0.556 (0.502-0.604) | 1.923 (1.769-2.080) | 3.050 (2.650- 3.464) | 18,817 |
| Senen | 0.105 (0.096-0.114) | 0.613 (0.580-0.640) | 2.057 (1.950-2.156) | 3.387 (3.108- 3.643) | 21,710 |
| Setiabudi | 0.115 (0.102-0.127) | 0.646 (0.601-0.681) | 2.165 (2.004-2.307) | 3.665 (3.250- 4.029) | 14,558 |
| Taman Sari | 0.103 (0.095-0.111) | 0.603 (0.575-0.631) | 2.091 (1.994-2.195) | 3.509 (3.251- 3.787) | 24,062 |
| Tambora | 0.152 (0.143-0.161) | 0.745 (0.723-0.764) | 2.611 (2.493-2.728) | 4.826 (4.527- 5.121) | 44,200 |
| Tanah Abang | 0.099 (0.078-0.118) | 0.59 (0.506-0.654) | 1.950 (1.723-2.168) | 3.092 (2.515- 3.646) | 14434 |
| Tanjung Priok | 0.118 (0.111-0.124) | 0.654 (0.631-0.673) | 2.136 (2.054-2.209) | 3.578 (3.367- 3.764) | 16850 |
| Tebet | 0.138 (0.131-0.144) | 0.711 (0.693-0.727) | 2.448 (2.364-2.532) | 4.365 (4.155- 4.574) | 22167 |

### Maps of subdistrict locations, spatial autocorrelation & annual subdistrict transmission intensity

**Figure S4. Map of Jakarta province with subdistrict and region locations.**

**
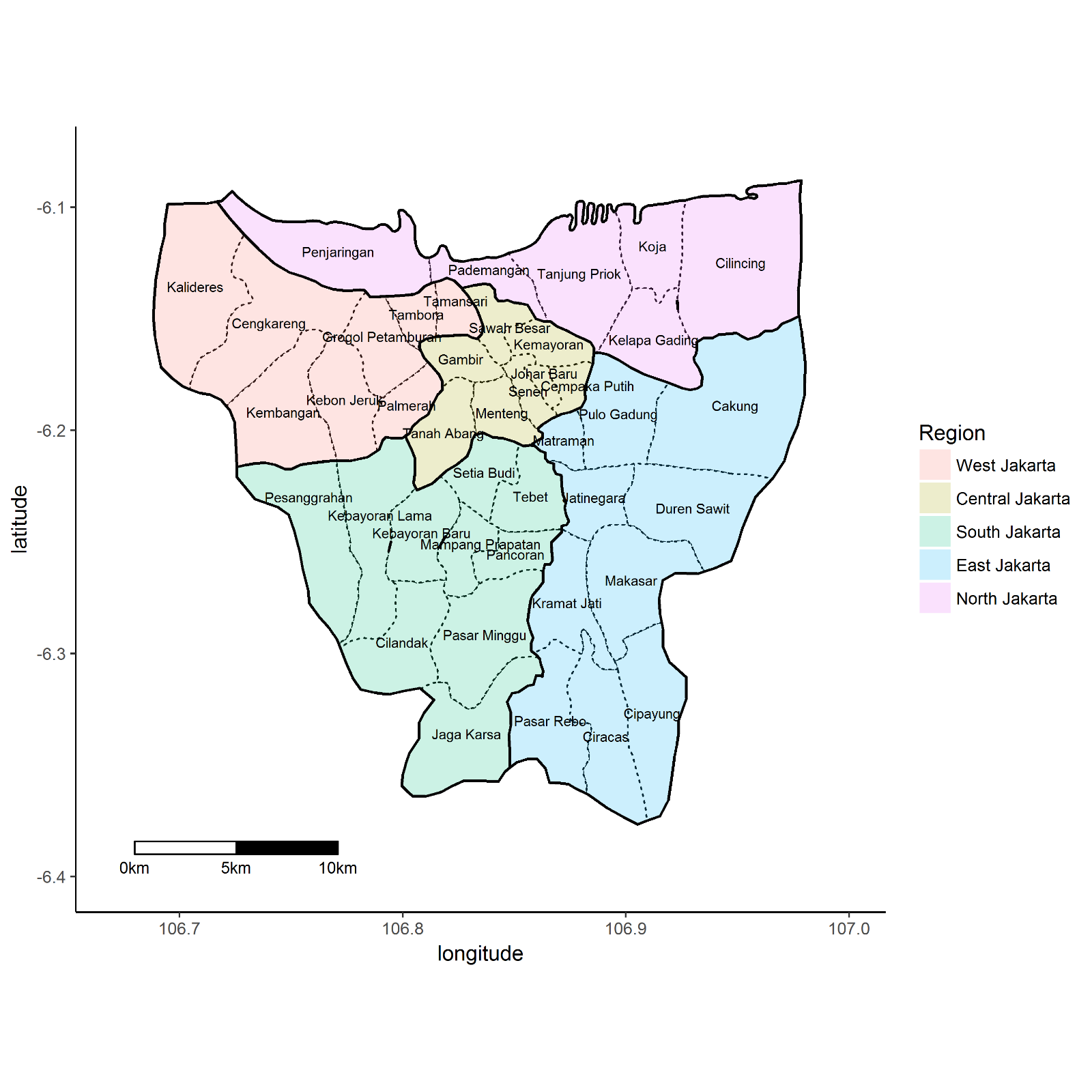
**

**Figure S5. Map of local indicators of spatial association (LISA) for subdistrict total force of infection (4λ) estimates. Red colour indicates spatial clustering of median transmission intensity estimates obtained from the fit of model variant PS to cumulative (2008-2017) age-stratified DHF incidence data reported in Jakarta’s subdistricts.**


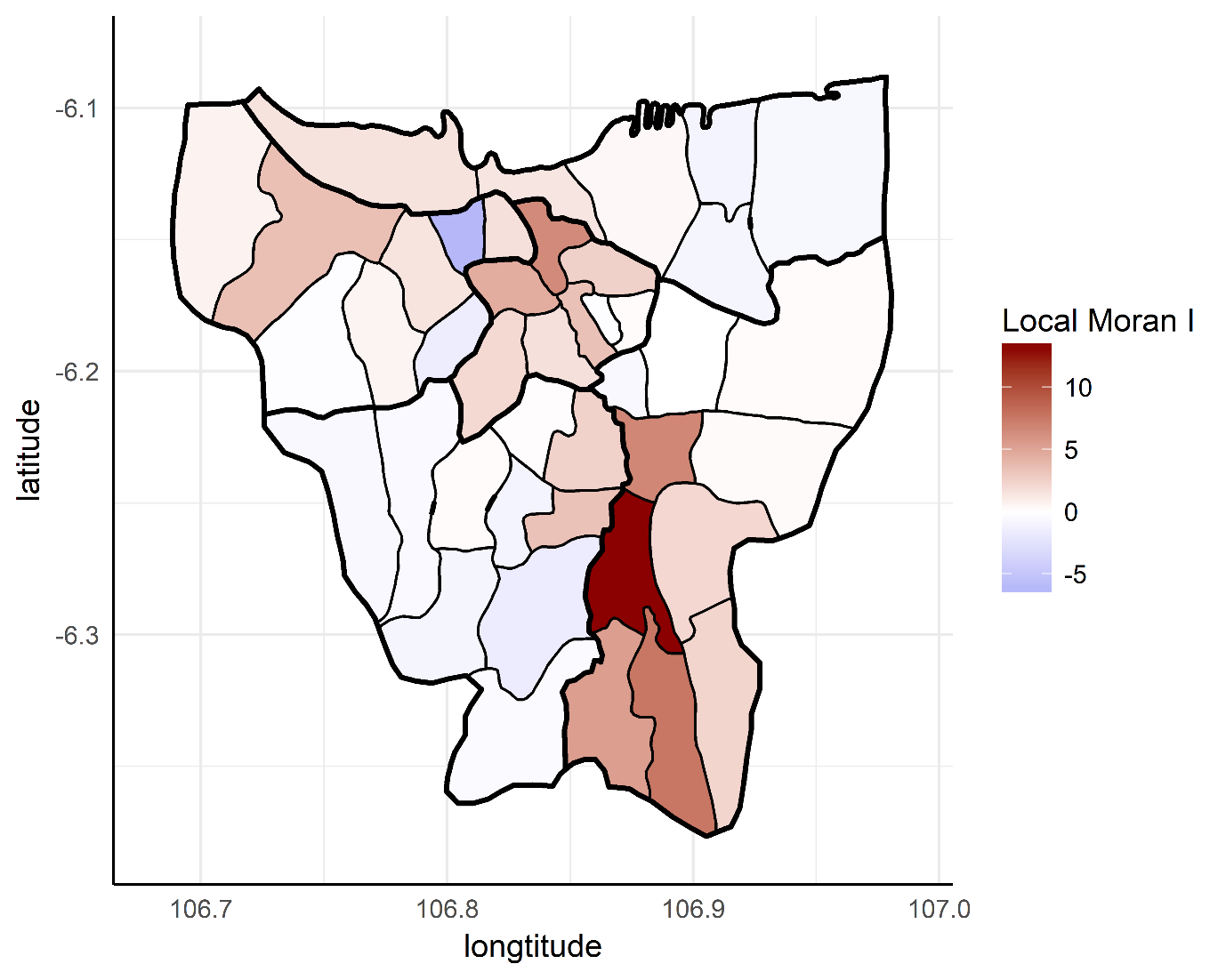


**Figure S6.** **Maps of the median total transmission intensity (4λ) estimates obtained from the fit of model 2 variant *PS* to yearly age-stratified DHF incidence rates reported between 2008 and 2017 in Jakarta’s subdistricts.** **For illustrative purposes we capped the colour scale at 30% force of infection, though some estimates were higher in individual years. The 2008 estimates represent long-term average total transmission intensity (force of infection) estimates for the period 1928-2008.**


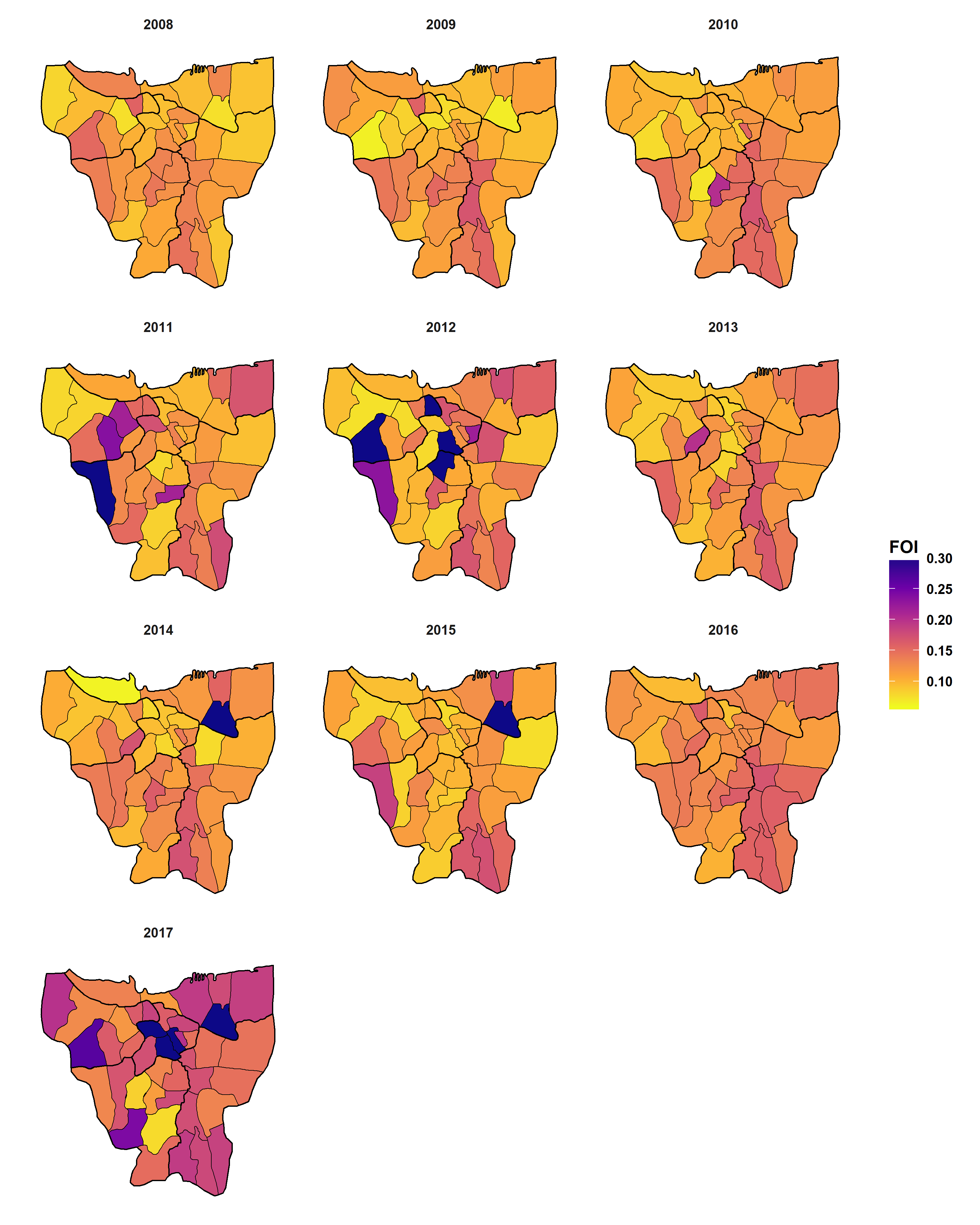


**Figure S7. Total transmission intensity (4λ) estimates, median and 95% credible intervals, obtained from the fit of model 2 variants *S* (orange) and *PS* (green) to yearly age-stratified DHF incidence rates reported between 2008 and 2017 in Jakarta’s subdistricts. All yearly parameter estimates are available in S1 Supplementary File.**


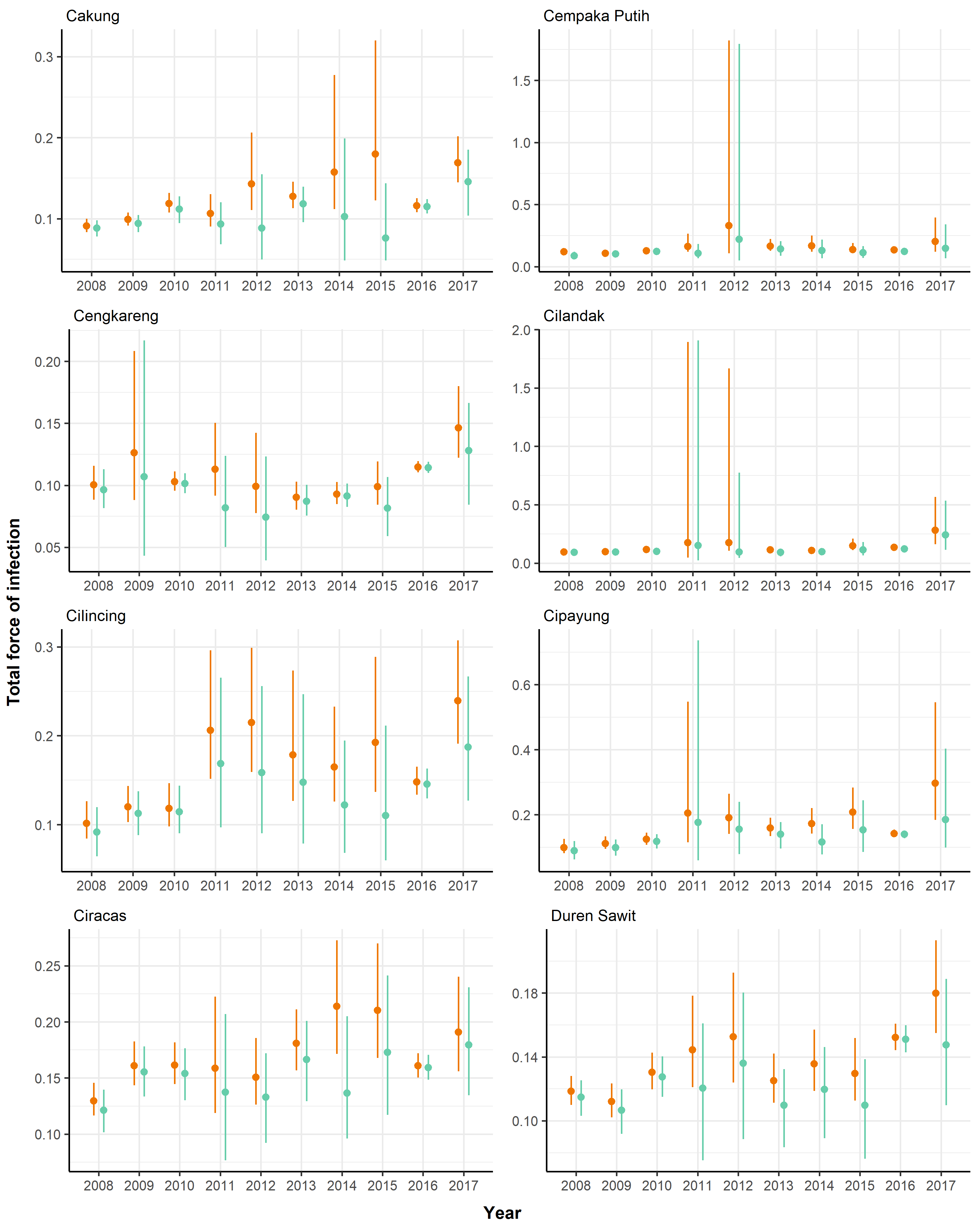


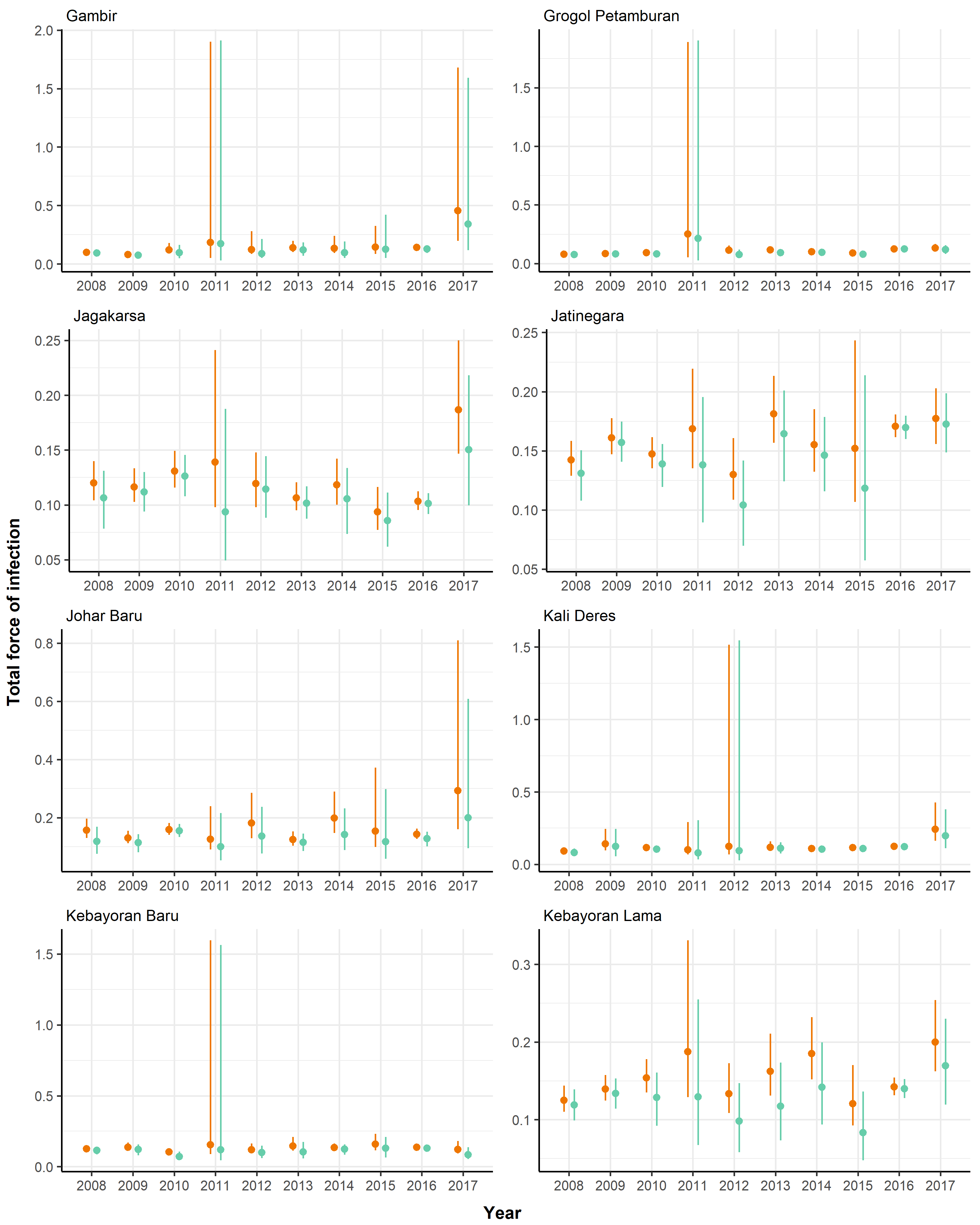


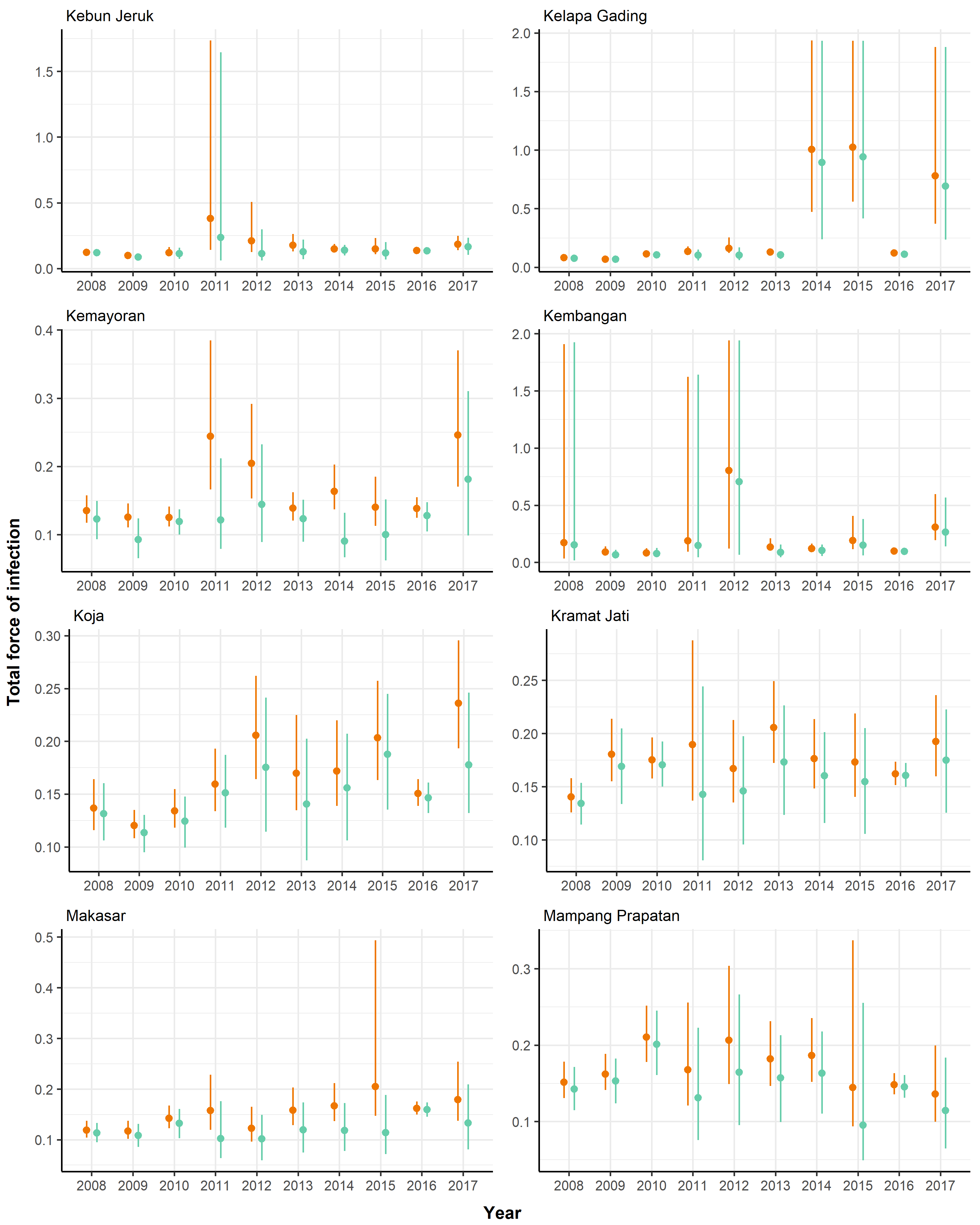


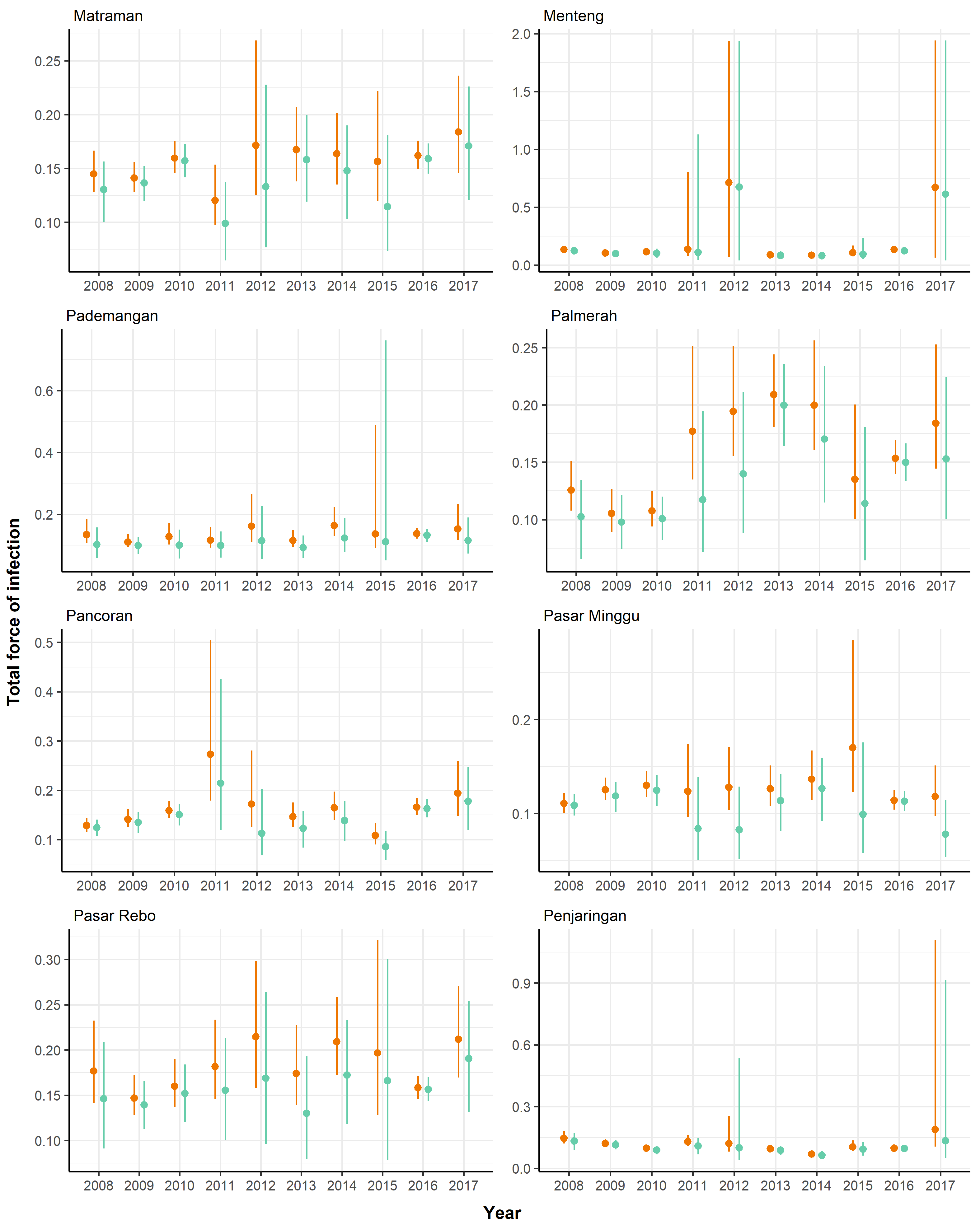


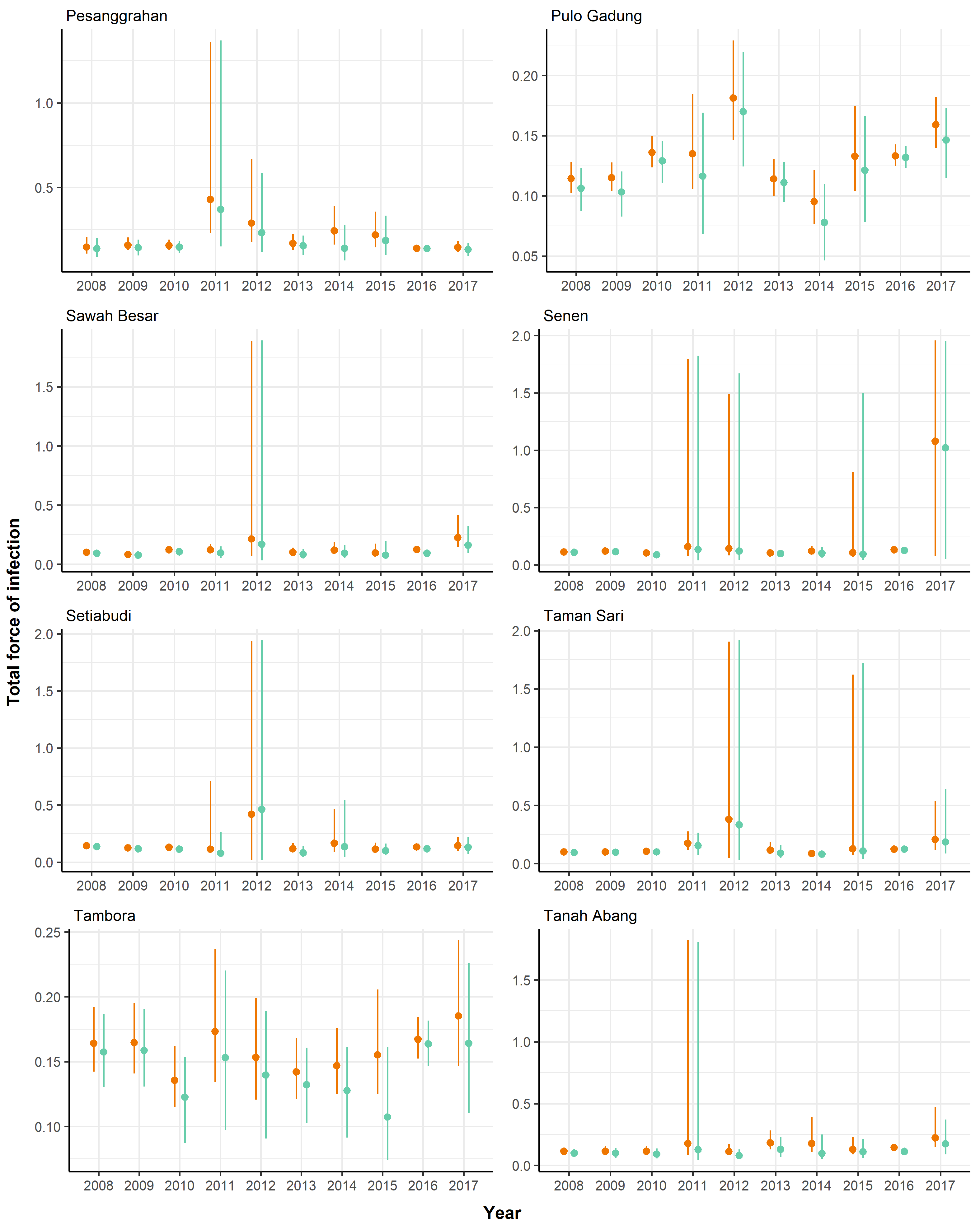


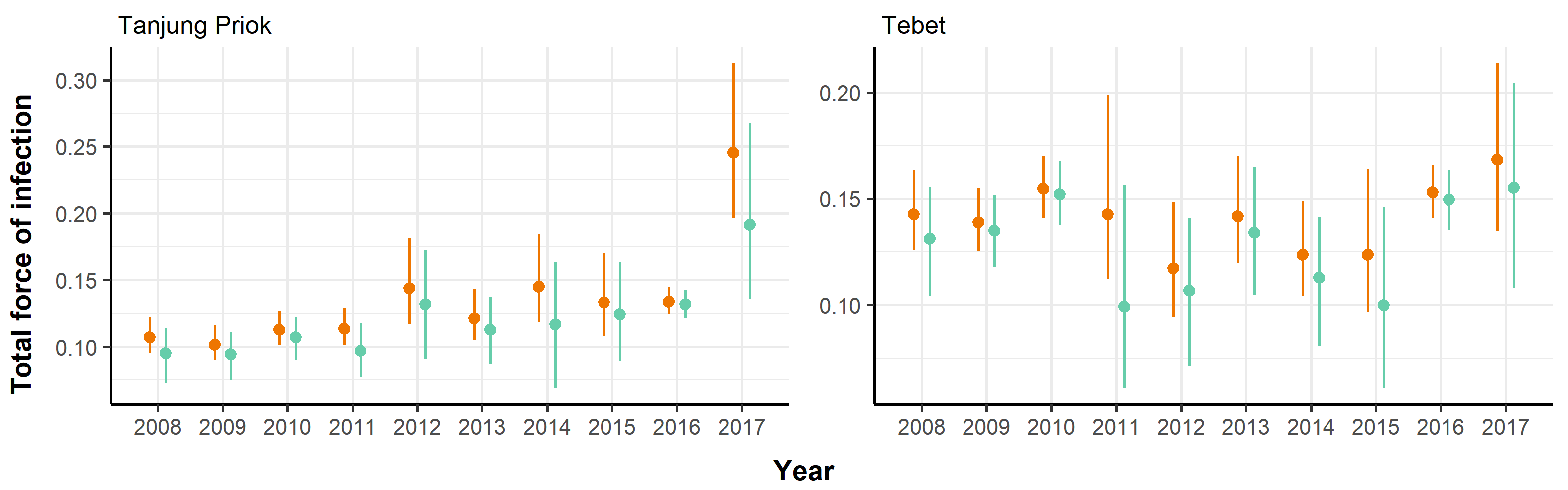


**Figure S8. Mean (blue line) and 95% confidence interval (grey shading) of linear regression model between average annual subdistrict total force of infection (4λ) estimates (obtained from the fit of model 1 variant *PS* to cumulative age-stratified DHF incidence rates reported in 2008-2017) and subdistrict population density estimates (available online (2)). Adjusted R^2^=0.049, P=0.086.**


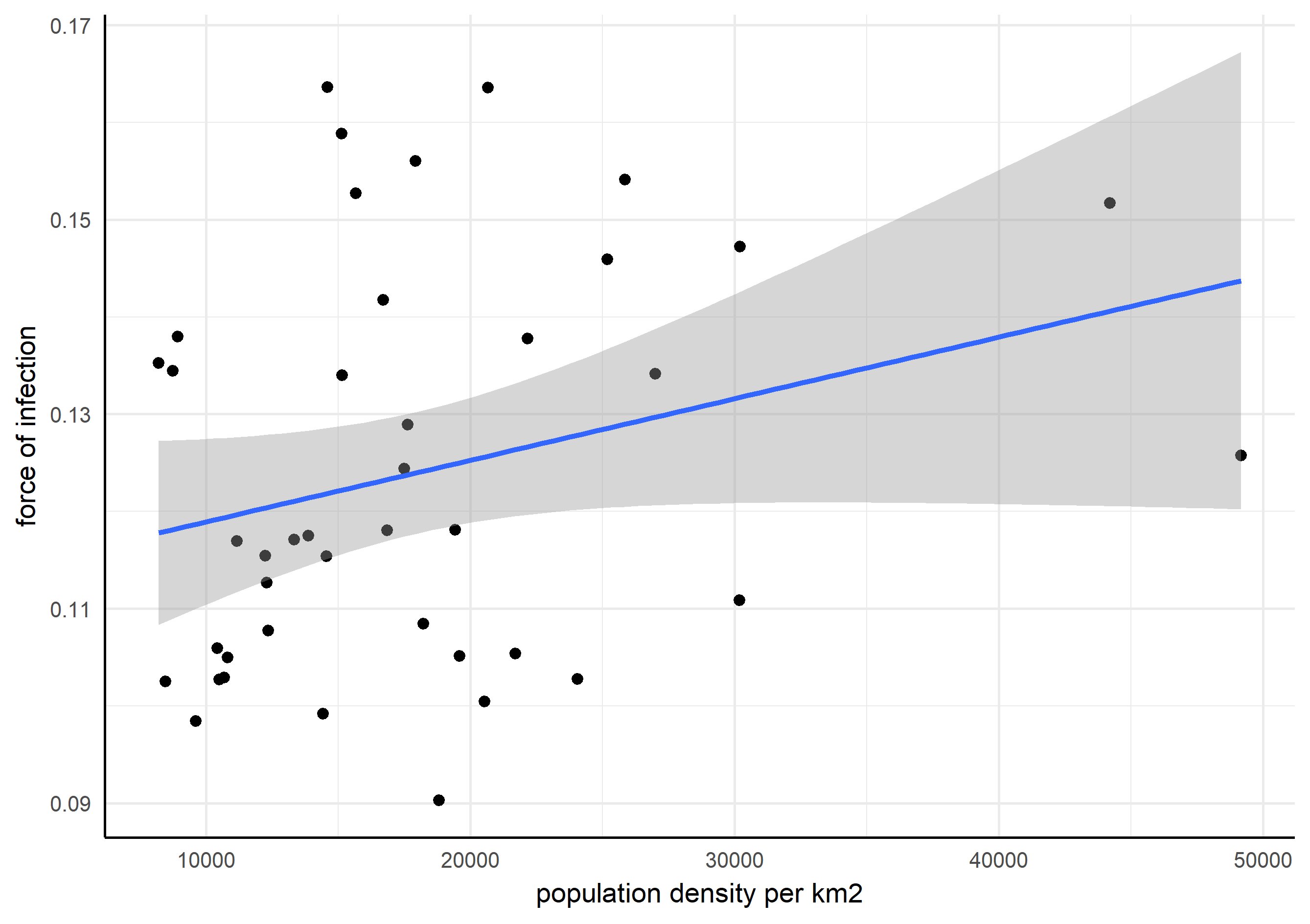


## References

1. Subdin Kesehatan Masyarakat, Seksi Survailans Epidemiologi [Internet]. [cited 2018 Feb 2]. Available from: http://www.surveilans-dinkesdki.net/

2. City Population Statistics [Internet]. [cited 2017 Jul 4]. Available from: http://www.citypopulation.de/Indonesia.html

## Supporting Information Legends

1. Supplementary file ‘S2.csv’ provides yearly subdistrict age-structures for the period 2008-2017.
2. Supplementary file ‘S3.csv’ provides yearly subdistrict parameter estimates obtained from Model 2.
