# Supplementary material for: Spatiotemporal variability in dengue transmission intensity in Jakarta, Indonesia
Source: PLoS Negl Trop Dis. 2020 Mar 6;14(3):e0008102. doi: 10.1371/journal.pntd.0008102 (PMC7080271; doi:10.1371/journal.pntd.0008102)
Supplement: Attachment — Submitted filename: Reviewers_responses_final.docx [file pntd.0008102.s004.docx]

**Point-by-point response to reviewers’ comments**

**Spatiotemporal Variability in Dengue Transmission Intensity in Jakarta, Indonesia**

Megan O’Driscoll, Natsuko Imai, Neil M. Ferguson, Sri Rezeki Hadinegoro, Hindra Irawan Satari, Clarence C. Tam, Ilaria Dorigatti

**Reviewer's Responses to Questions**

**Reviewer #1: (No Response)

Reviewer #2: Although I think the article has merit as a showcase of the application to Indonesian dengue control program, methodological concerns need to be addressed to ensure validity of the analyses.**

We thank the reviewer for their positive comment and for giving us the opportunity to address their concerns on the methodology adopted. Please find our responses below.

**I understand that there might be computing issues with complicated models. However, there are examples that changing model assumptions, i.e. over-dispersed distributions in this case, can alter results significantly. How would you justify your results if you cannot apply the over-dispersed models?**

In the discussion, we mention that a potential way to increase the uncertainty around the predictions could be to assume more flexible distributions that allow the variance to exceed the mean. However, higher flexibility comes at the price of additional unknown parameters and in our case, including over-dispersion makes convergence of the MCMC algorithm challenging – this not only hinders the estimation of the over-dispersion parameter, but also of all other parameters of the model.

When feasible, the use of over-dispersed models (or alternatively conjugate priors on model parameters) could be a potential way to inflate the uncertainty around the central estimates but is not expected to significantly alter the central estimates nor the fit to the data. The issue of MCMC producing highly precise estimates when calibrating complex models and datasets is well known and rather common. Ultimately, computational issues (e.g. slow convergence) are important from a practical perspective and whilst we acknowledge that the estimates shown in the results may underestimate the actual uncertainty, over-dispersed distributions would not be expected to significantly alter the mean estimates nor the overall fit to the data.

The model applied in our analysis can accurately reproduce the highly heterogeneous age-distribution of the observed dengue case-notification data across Jakarta’s sub-districts, implying that the model structure and underlying assumptions adequately reflect the life history of the disease and the reporting process. We are confident in the results/estimates also because we thoroughly tested the model on simulated data before applying it to the real data (though we decided not to include our analysis on simulated data in the manuscript in order to keep the focus on the application to the Jakarta data and its public health implications).

Not only does our model successfully capture the average disease patterns observed across ages and subdistricts; in several instances, it also successfully reproduces the observed uncertainty around the data. On some occasions, however, our model (which assumes a Poisson and multinomial likelihood, and hence implicitly assumes that the variance does not exceed the mean) tends to underestimate the observed uncertainty around the data. We acknowledge this as a limitation of our analysis, as mentioned in the discussion, and have clarified that this can result in an underestimation of parameter uncertainty levels in lines 459-469, as follows: “*Additionally, the parameter results shown in this analysis are estimated assuming no overdispersion in the distribution of dengue cases (i.e. we assume a Poisson distribution for the total number of cases and a multinomial distribution for the age distribution of cases), which may result in an underestimation of the uncertainty around the central parameter estimates. In exploratory analysis, we tested the use of over-dispersed case distributions (specifically assuming a negative binomial distribution for the total number of cases and a Dirichlet multinomial likelihood for the age-distribution of cases). However, the use of over-dispersed distributions hindered MCMC convergence.*”.

**On page 10, it’s not clear from model 2 if the authors apply equations 3-6 independently for each subdistrict or as the whole province because the spatial index is not specified. It’s also evident from spatial association metrics that spatial correlation is present in the data but does not seem to be captured in model 2.**

The reviewer raises a valid point. In our analysis we explored the spatial association of the force of infection estimates rather than the spatial correlation in the case-notification data, as these latter do not necessarily reflect dengue transmission intensity patterns.

It is correct to say that model 2 was fitted independently to each subdistrict and does not account for any spatial interactions. We have now clarified this in lines 252-254 as follows: “*Model 2 was fitted independently to each geographic unit (i.e. at the province- and subdistrict-level, separately), assuming no spatial interactions.*”.

Accounting for spatial structure would largely increase the dimensionality of the inferential problem, as we would need to infer the neighbourhood structure among Jakarta’s subdistricts on top of the temporal dependencies, which are explicitly accounted for in the model. Due to the large number of subdistricts, it would be challenging to simultaneously infer the model parameters and the spatial structure of a fully explicit spatiotemporal model. We have commented on this additional model limitation in lines 453-456 as follows: “*While we explicitly accounted for temporal trends, we assumed no spatial interactions among Jakarta subdistricts. Accounting for spatial structure would increase the complexity of the inferential problem and make parameter inference challenging.*”.

Spatial clustering was investigated using the estimates obtained from Model 1, which produces long-term average estimates of transmission intensity, as longer-term hotspots of transmission are more relevant for control efforts and policy decisions.

**Line 254, the authors assumed uniform priors on all parameters. Please provide the range of the priors.**

Thank you for the suggestion. We have now included in the main text and in the SI the range of all priors (from 0 to 1).

--------------------

**Reviewer #1: (No Response)

Reviewer #2: I like that the authors list out the study limitations. However, I wonder how realistic and useful this work would be for the dengue control program under those assumptions. For example, the authors allow for post-secondary infection to contribute to transmission which do not cause severe conditions. This is quite a strong assumption and difficult to justify. Since the authors are working with policy makers, that would be more useful to the control program to have results under different assumptions rather than just one instance presented to the government.**

We agree with the reviewer that results using different plausible assumptions should be reported in order to understand the uncertainty of the results. Indeed, we do show results from different model assumptions, which we elaborate on below. We believe that the confusion surrounding this derived from a sentence in the discussion (lines 437-439) where we originally stated “*Further, though we allow post-secondary infections to contribute to transmission, we assumed that they do not cause severe disease*.” In this sentence we implicitly referred to one of the assumptions (assumption 1) used in the R0 calculation. However, we do not allow post-secondary infections to contribute to transmission when estimating transmission intensity which we appreciate was not clear from this sentence. We have corrected the sentence to “*Further, we assumed that post-secondary infections do not cause severe disease and are hence not detected*”.

In our analysis we have explored two different assumptions on the occurrence of symptomatic infections: in model S we assumed that only secondary infections cause dengue disease and in model PS we assumed that both primary and secondary infections cause dengue disease. As expected, allowing primary infections to contribute to disease (model PS) produces lower transmission intensity estimates than when assuming that only secondary infections cause disease (model S).

In additional analyses shown in the supplementary information, we derive estimates of the basic reproduction number from our force of infection estimates. Here, we explored two different assumptions on the contribution of post-secondary infections to transmission (see SI section 3.4). Under assumption 1, we assumed that all infections (primary-quaternary) contribute to transmission and are equally infectious, with no cross-immunity between serotypes. Under assumption 2 we assumed that only primary and secondary infections are infectious and contribute to onward transmission. These estimates are shown in Table S5, section 4.2 of the Supplementary information. It was these assumptions that we referred to in the discussion but have now corrected to avoid confusion.

**Reviewer #1: The authors present an approach for estimating the force of infection from age-stratified case reports. The manuscript is well written and the methods and results are clearly described. The findings are presented in light of dengue vaccination campaigns (among others), where estimates of the pre-exposed population at a certain age are important for the implementation of test-before-vaccination approaches. The manuscript is of good quality and I recommend it for publication. I only have a few minor questions and suggestions:**

Thank you for the positive feedback and constructive suggestions. Please find our point-by-point responses to the comments below.

**1. The authors estimate reporting and misreporting rates. It would be worthwhile to report and discuss these in the manuscript. How to the estimated reporting rates differ between primary and secondary infections? How do estimates compare with published estimates (for instance from JD Stanaway et al, 2016, Lancet)?**

The difference in reporting rates for primary and secondary infections is reported in lines 301-305 – “*This same model produced estimates of the probability of a dengue infection resulting in a reported hospitalized DHF case (reporting rate for secondary infections, ρ) of 0.077 (95%CrI: 0.076-0.078), with primary infections being 0.002 (95%CrI: 0.001-0.009) times as likely to result as hospitalized DHF (reporting probability, γ1 relative to ρ) (SI, Table S4)*.”. We have also added further detail on the variability in reporting rates over time in lines 396-398, as follows: “*Annual reporting probabilities varied by year, from 0.024 (95%CrI: 0.022-0.026) in 2017 to 0.179 (95%CrI: 0.176-0.182) in 2016, the year in which a significant outbreak took place.*”.

Unfortunately, our reporting probabilities are not directly comparable to the work of JD Stanaway et al, 2016, as they estimate expansion factors for the under/over reporting of symptomatic dengue cases. The estimates shown in our analysis are the combined probability that a dengue infection becomes hospitalized in addition to becoming a reported dengue case. We have now added some detail (lines 398-400) on the comparability of our estimates to those observed during a prospective cohort study of dengue infections in children aged 4-16 in Bangkok, in which 12.5% of secondary infections observed resulted in hospitalization, as follows: “*Similar estimates have been observed in a prospective cohort study of dengue infections in Thai children aged 4-16, where 12.5% of secondary infections resulted in hospitalized dengue cases (28).”.*

**2. The authors report hotspots of infection, i.e., areas where transmission is consistently higher than elsewhere. One might expect that, after a big outbreak year, the force of infection in such hotspots is lower due to build up immunity. Understanding such mechanisms could be helpful for targeting control efforts. Do the authors see any such pattern? Is the ranking across sub-districts stable over the years?**

We agree with the reviewer that the ranking of high transmission intensity subdistricts is expected to vary over time due to the build-up of immunity and other transient factors such as local climate and other conditions. Indeed, we do see variability in the ranking of subdistrict transmission intensity over time as shown in supplementary figure S6. Whilst we agree with the reviewer that understanding the mechanisms that drive changes in transmission intensity over time is crucial, disentangling the mechanisms of multiple potential drivers (local weather, vector suitability, socio-economic factors, immunity, etc.,) is difficult and not explored in this analysis. We chose only to investigate areas of long-term clustering transmission in order to highlight regions of consistently high transmission intensity, where control efforts may be of most need. We have highlighted and clarified in the discussion (lines 386-388) that the ranking of subdistrict transmission intensity varies by year and that the clustering of low and high transmission intensity refers to long-term average estimates. Lines 386-388 now read as follows: “*Though ranking of subdistrict transmission intensity estimates varied by year, our analysis identified a long-term average hot-spot of dengue transmission in the southeast of Jakarta province and clustering of low transmission intensity in the region of Central Jakarta*”.

**3. I am not sure all state variables are described in the text. (M is equation 4, maybe others). Please check if all are included.**

We thank the reviewer for noticing this. We have now included a description of state variables in lines 237-241 as follows: “*The incidence of primary and secondary infections in subsequent years can then be calculated based on the estimated population-level immunity profiles, i.e.* *the estimated proportion of the population susceptible, S, and the estimated proportion of the population exposed to a monotypic infection, M, by age, as shown in equations 3-6*”.

**Reviewer #2: (No Response)**
